# HSDL2 links nutritional cues to bile acid and cholesterol homeostasis

**DOI:** 10.1126/sciadv.adk9681

**Published:** 2024-05-31

**Authors:** Nolwenn Samson, Cristina R. Bosoi, Christian Roy, Laurie Turcotte, Laura Tribouillard, Mathilde Mouchiroud, Line Berthiaume, Jocelyn Trottier, Heitor C. G. Silva, Thomas Guerbette, Ana Belén Plata-Gómez, Aurèle Besse-Patin, Alicia Montoni, Nicolò Ilacqua, Jennifer Lamothe, Yemima R. Citron, Yves Gélinas, Stéphane Gobeil, Roberto Zoncu, Alexandre Caron, Mathieu Morissette, Luca Pellegrini, Patrick J. Rochette, Jennifer L. Estall, Alejo Efeyan, Michael Shum, Étienne Audet-Walsh, Olivier Barbier, André Marette, Mathieu Laplante

**Affiliations:** ^1^ Centre de Recherche de l'Institut universitaire de cardiologie et de pneumologie de Québec (CRIUCPQ), Université Laval, Québec, QC, Canada.; ^2^ Centre de recherche sur le cancer de l’Université Laval, Université Laval, Québec, QC, Canada.; ^3^ Centre de recherche du Centre Hospitalier Universitaire (CHU) de Québec-Université Laval, Axe Endocrinologie et néphrologie, Québec, QC, Canada.; ^4^ Faculté de médecine, Université Laval, Québec, QC, Canada.; ^5^ Metabolism and Cell Signaling Laboratory, Spanish National Cancer Research Centre (CNIO), Madrid, Spain.; ^6^ Institut de recherches cliniques de Montréal (IRCM), Montréal, QC, Canada.; ^7^ Axe Médecine régénératrice, Centre de Recherche du CHU de Québec-Université Laval, Hôpital du Saint-Sacrement, Québec, QC, Canada.; ^8^ Centre de recherche CERVO, Québec, QC, Canada.; ^9^ Department of Molecular and Cell Biology, University of California, Berkeley, Berkeley, CA, USA.; ^10^ Innovative Genomics Initiative at the University of California, Berkeley, Berkeley, CA, USA.; ^11^ Faculté de Pharmacie, Université Laval, Québec, QC, Canada.; ^12^ Department of Biochemistry, Microbiology and Immunology, Faculty of Medicine, University of Ottawa, Ottawa, QC, Canada.; ^13^ Département d’Ophtalmologie et ORL – chirurgie cervico-faciale, Université Laval, Québec, QC, Canada.; ^14^ Faculté de médecine, Université de Montréal, Montréal, QC, Canada.

## Abstract

In response to energy and nutrient shortage, the liver triggers several catabolic processes to promote survival. Despite recent progress, the precise molecular mechanisms regulating the hepatic adaptation to fasting remain incompletely characterized. Here, we report the identification of hydroxysteroid dehydrogenase–like 2 (HSDL2) as a mitochondrial protein highly induced by fasting. We show that the activation of PGC1α-PPARα and the inhibition of the PI3K-mTORC1 axis stimulate HSDL2 expression in hepatocytes. We found that HSDL2 depletion decreases cholesterol conversion to bile acids (BAs) and impairs FXR activity. HSDL2 knockdown also reduces mitochondrial respiration, fatty acid oxidation, and TCA cycle activity. Bioinformatics analyses revealed that hepatic *Hsdl2* expression positively associates with the postprandial excursion of various BA species in mice. We show that liver-specific HSDL2 depletion affects BA metabolism and decreases circulating cholesterol levels upon refeeding. Overall, our report identifies HSDL2 as a fasting-induced mitochondrial protein that links nutritional signals to BAs and cholesterol homeostasis.

## Teaser

HSDL2 is a mitochondrial protein that controls bile acid and lipid metabolism in response to nutritional cues.

## INTRODUCTION

The liver controls several functions in mammals including lipid and glucose metabolism, cholesterol and bile acid (BA) synthesis, lipoprotein secretion, ketone body production, protein and amino acid metabolism, and breakdown of xenobiotic compounds (*1*). Most of the metabolic functions fulfilled by the liver are carried out by the hepatocytes, which constitute the main cell type of this organ. Studies performed over the past decades have linked hepatocyte dysfunction to the development of several pathological conditions including fatty liver disease, dyslipidemia, glucose intolerance, insulin resistance, and cardiovascular diseases (*2*).

Hepatocytes are extremely sensitive to circulating nutrients and hormones, and their dynamic adaptation to nutritional cues is essential for the maintenance of systemic glucose and lipid homeostasis. In response to feeding, the rise in glucose and insulin levels triggers anabolism in hepatocytes (*3*). In the liver, insulin activates phosphoinositide 3-kinase (PI3K) and mechanistic target of rapamycin complex 1 (mTORC1) to stimulate anabolic processes such as glucose uptake, glycogen synthesis, and lipogenesis (*4*, *5*). Activation of insulin signaling also represses catabolic processes including gluconeogenesis, glycogen breakdown, and ketogenesis. During the transition to fasting, the decrease in circulating nutrients and insulin is sensed by the liver, which allows an efficient transition from anabolism to catabolism. Promoting catabolism in hepatocytes is essential to activate hepatic glucose production and ketogenesis, two processes providing energy to support the activity of the brain and peripheral tissues during periods of low nutrients and energy availability (*6*).

Transcriptomics experiments showed that deep changes in gene expression rapidly take place in the liver during the feeding/fasting transition (*7*–*12*). Several nuclear receptors were identified to play roles in regulating the hepatic response to nutritional challenges (*6*). Seminal studies identified peroxisome proliferator–activated receptor alpha (PPARα) and PPAR gamma coactivator 1 alpha (PGC1α) as key transcriptional regulators promoting hepatic gene expression in response to fasting (*9*, *13*–*16*). This protein complex, which is activated by fatty acids, controls the expression of numerous genes promoting lipid oxidation and energy production in the liver. Other nuclear receptors including farnesoid X receptor (FXR) and liver X receptor (LXR) have also been shown to control hepatic gene expression by sensing cholesterol derivates such as BAs and oxysterols (*17*). The complex interplay among these regulators not only guarantees optimal metabolic adaptation during fasting but also prepares the liver to respond efficiently to food intake.

Despite progress in the understanding of the molecular mechanisms regulating the hepatic adaptation to fasting, the full picture of the catabolic processes triggered in hepatocytes upon nutritional deprivation remains incompletely characterized. Here, using an original screening approach, we report the identification of hydroxysteroid dehydrogenase–like 2 (HSDL2) as a protein affecting hepatic catabolism upon fasting. HSDL2 localizes to the mitochondria and its expression is highly induced by fasting. Studies performed in vitro and in vivo revealed that fasting-associated activation of PGC1α-PPARα plays a central role in driving HSDL2 expression. Conversely, we show that insulin repressed HSDL2 levels through a PI3K-mTORC1–dependent mechanism. Supporting a role for HSDL2 in regulating cellular catabolism, we found that HSDL2 knockdown impaired mitochondrial respiration, fatty acid oxidation, and tricarboxylic acid cycle (TCA) activity in hepatocytes. Specifically, we found that HSDL2 depletion decreased cholesterol conversion to BAs and impaired the expression of numerous genes under the control of FXR, the primary BA receptor in mammals. Bioinformatics analyses revealed that hepatic *Hsdl2* expression positively associates with the postprandial excursion of various BA species in mice. In line with these results, we found that liver-specific depletion of HSDL2 alters hepatic BA metabolism and reduces circulating cholesterol upon refeeding. Our study positions hepatic HSDL2 as a metabolic regulator that affects systemic adaptation to nutritional cues.

## RESULTS

### HSDL2 is a protein highly expressed in catabolic hepatocytes

To uncover mechanisms regulating catabolism in hepatocytes, we took advantage of a phenotypic screening approach that was previously used to identify regulators of fat cell development (*18*, *19*). This approach is based on the premise that cellular heterogeneity is a fundamental attribute of all cell populations and that any given phenotype usually follows a normal distribution. Briefly, it was reported that by performing limiting dilutions of a parental cell line, it is possible to derive subclonal lines presenting extremely divergent phenotypes and that these cells can serve as a powerful discovery platform to identify genes regulating specific biological processes (fig. S1A).

**Fig. 1.**
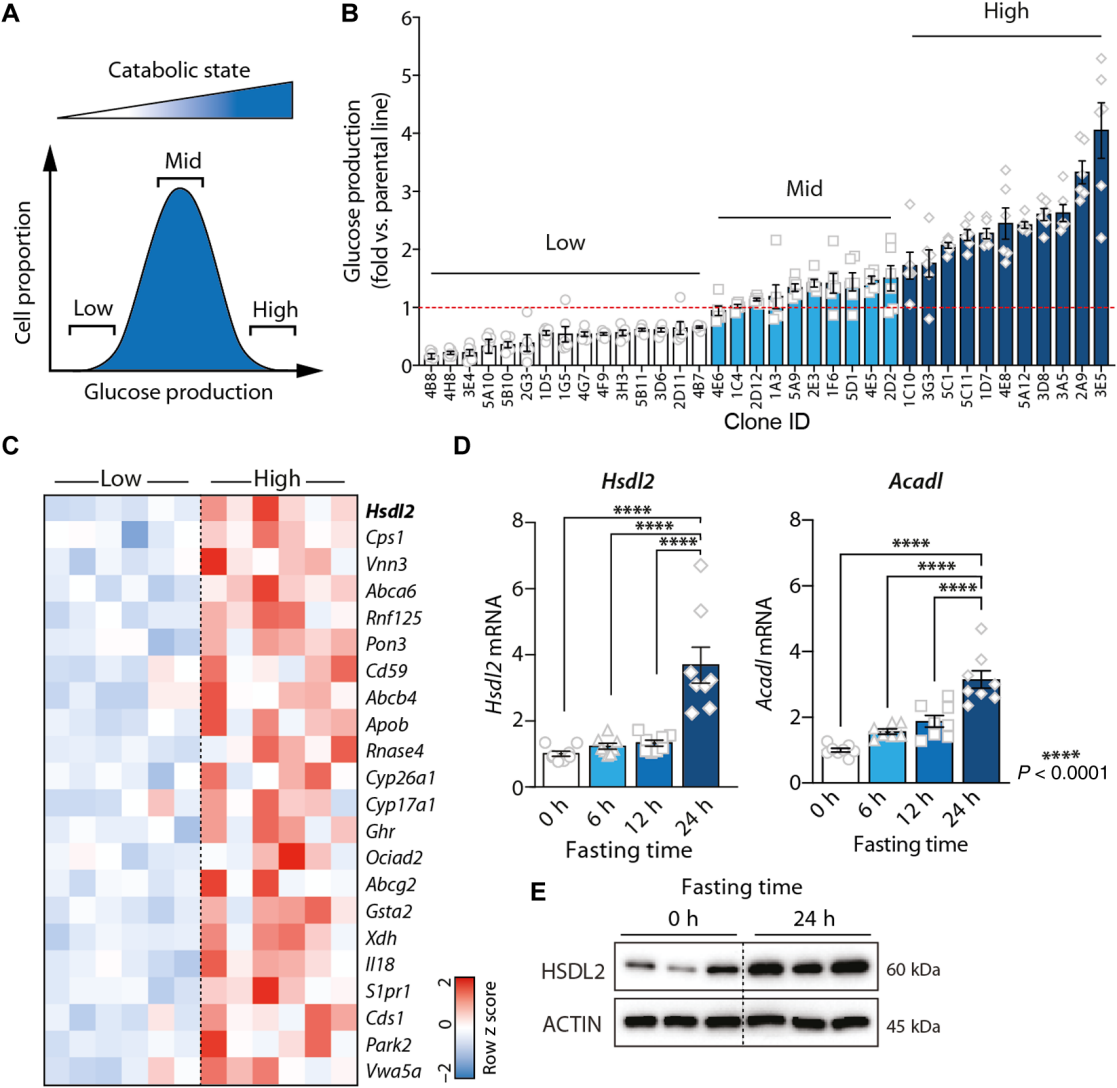
HSDL2 is a protein highly expressed in catabolic hepatocytes. (**A**) Glucose production is a catabolic process that is normally distributed in cultured hepatocytes. This characteristic was exploited to identify genes controlling catabolism in hepatocytes. (**B**) Glucose production was measured in 36 clonal lines isolated from a parental culture of FAO cells. Glucose was measured in six independent wells per clone, and results were corrected by protein content. (**C**) Heatmap showing the expression levels of selected genes measured by microarray in Low (*n* = 6) and High (*n* = 6) catabolic cell lines. The genes presented are the 22 high-priority candidate genes identified using the decision matrix presented in fig. S1E. (**D**) Male C57BL/6J mice were euthanized after either 0, 6, 12, or 24 hours (*n* = 8 per group) of fasting, and liver samples were collected. RNA was extracted, and gene expression was measured by quantitative real-time polymerase chain reaction (RT-qPCR). (**E**) Protein lysates were prepared from liver samples collected from mice fasted for 0 or 24 hours, and Western blots were performed for the indicated proteins. Representative samples are shown (*n* = 3 per group). In all panels, data are presented as means ± SEM. In (D), significance was determined by one-way analysis of variance (ANOVA) with Tukey’s multiple-comparisons test.

Because glucose production is a key metabolic pathway triggered in catabolic hepatocytes and because this process is simple to assess in vitro, glucose production was chosen as a proxy to isolate cells with different catabolic rates (Fig. 1A). Single cells were isolated and amplified from a parental culture of FAO cells, a well-established rat hepatoma cell line commonly used to study hepatocyte metabolism in vitro. Overall, a total of 36 subclonal lines were derived (fig. S1B). Notably, we found that the catabolic rate of each clone was highly variable, as illustrated by the different gluconeogenic potential of each line (Fig. 1B). To characterize the gene signature defining the catabolic capability of these cells, transcriptomics analyses were performed using Low and High glucose-producing lines. A total of 608 genes were found to be differentially expressed between these two groups (table S1). Confirming the validity of our approach, comprehensive analysis of this gene signature with Metascape (*20*) identified “Metabolic process” as the most significant gene ontology parent pathway affected between Low and High lines (fig. S1C and table S2). Specifically, “Monocarboxylic acid metabolic process,” “Steroid metabolic process,” and “Metapathway biotransformation phase I and II” were the three most significant processes identified in this analysis (fig. S1D and table S3).

We then constructed a simple decision matrix to define a high-priority gene set that may reveal insights into the mechanisms controlling catabolism in hepatocytes. An overview of the decision tree and the results from this analysis are presented in fig. S1E and table S4. Overall, 22 genes stood out from this analysis, including some previously reported to play key metabolic roles in hepatocytes (e.g., *Cps1*, *Apob*, and *Park2*; Fig. 1C). We found that the top-priority candidate was a poorly characterized gene named *Hsdl2*. Previous studies reported that HSDL2 levels are high in several cancers and that repressing HSDL2 impairs proliferation and survival (*21*–*30*). However, the mechanisms behind these effects remain unclear, and the precise metabolic functions of HSDL2 in hepatocytes were never investigated. Here, we found that *Hsdl2* levels are elevated in the High versus Low catabolic lines (Fig. 1C and fig. S1F). Moreover, we observed that hepatic *Hsdl2* mRNA and HSDL2 protein levels were strongly increased in response to food deprivation in mice, similarly to long-chain specific acyl–coenzyme A (CoA) dehydrogenase, mitochondrial (*Acadl*), a gene previously reported to be induced by fasting (Fig. 1, D and E) (*31*). *Hsdl2* levels peaked after 24 hours of fasting, when hepatic glycogen stores were already depleted and gluconeogenic markers were already fully activated, suggesting that HSDL2 might participate in the late response to food deprivation (fig. S1, G and H). Together, these results identify HSDL2 as a protein highly expressed in catabolic hepatocytes.

### HSDL2 is a mitochondrial protein

**Fig. 2.**
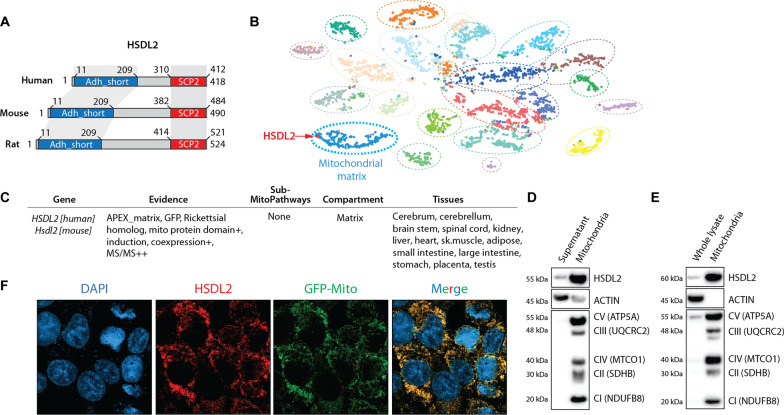
HSDL2 is a mitochondrial protein. (**A**) Schematic presentation of human, mouse, and rat HSDL2 protein. The conserved domains are presented in red and blue. (**B**) Visual presentation of subcellular HSDL2 localization in HEK293 cells using the Cell Map Resource. In this graph, each dot represents a protein, and each dashed circle represents a cell compartment. (**C**) Presentation of the results of HSDL2 localization using the MitoCarta 3.0 resource. Results are presented for both human and mouse HSDL2. A complete description of the “evidence” sections follows. APEX_matrix, detected in the mitochondrial matrix in HEK293 cells based on APEX labeling. GFP, mitochondrial localization observed by green fluorescent protein (GFP)–tagging and low-resolution microscopy in this study (*95*). Rickettsial homolog, gene has a homolog to *Rickettsia prowazekii* based on BLASTP (with expected <1 × 10^−3^) or jackHMMER reciprocal hit. Mito protein domain, protein has a mitochondrial-specific Pfam domain. Coexpression, mouse transcript is coexpressed with known mitochondrial genes across mouse tissues (+ and ++ indicate higher levels of coexpression). MS/MS, mouse protein detected with high confidence in mitochondrial samples from 14 tissues (+ and ++ indicate higher confidence of detection). (**D**) HepG2 cells were fractionated to separate mitochondria from the other organelles. Protein lysates were prepared from mitochondrial pellet and the supernatant containing nonmitochondrial organelles. Western blots were next performed for the indicated proteins. (**E**) Mitochondria were isolated from mouse liver samples, and Western blots were performed, as described in (D). (**F**) HepG2 cells expressing a GFP-tagged mitochondrial protein (green) were fixed, and immunofluorescence assay was performed against endogenous HSDL2 (red). Nuclei were stained with 4′,6-diamidino-2-phenylindole (DAPI; blue). The scale presented is 101.41 by 101.41 μm. Representative pictures are shown.

HSDL2 is a member of the short-chain dehydrogenase/reductase (SDR) family that is highly conserved in evolution (fig. S2A). HSDL2 contains an N-terminal short-chain alcohol dehydrogenase (ADH) domain and a C-terminal sterol carrier protein 2 (SCP2) domain, two structural features that participate respectively in nicotinamide adenine dinucleotide (oxidized form) (NAD)/NAD phosphate (NADP)–dependent oxidoreduction reactions and in the binding of sterols and other lipids (Fig. 2A and fig. S2A) (*32*, *33*). To gain insight into the metabolic functions of HSDL2, we first investigated its cellular localization using various approaches. We first used the Human Cell Map database, a BioID-based resource that provides the intracellular locations of thousands of unique proteins in human embryonic kidney (HEK) 293 cells (*34*). This analysis revealed that HSDL2 resides in the mitochondrial matrix (Fig. 2B). In addition, the MitoCarta3.0 resource, which inventories human and mouse mitochondrial proteins (*35*), shows that HSDL2 localizes to the mitochondrial matrix in 14 different tissues, including the liver (Fig. 2C). In line with these results, we found that the N-terminal region of human and mouse HSDL2 contains a mitochondrial targeting sequence that predicts mitochondrial localization (fig. S2, B and C) (*36*). To further confirm these findings, mitochondria were isolated from human HepG2 cells and mouse liver, and HSDL2 levels were measured by immunoblotting. As predicted, we observed that HSDL2 was enriched in the mitochondrial fractions in these two models (Fig. 2, D and E). Complementary immunofluorescence assays confirmed that endogenous HSDL2 also localizes to the mitochondria in vitro (Fig. 2F). Together, these findings convincingly demonstrate that HSDL2 is a mitochondrial protein.

### The expression of *Hsdl2* is induced by PGC1α-PPARα

**Fig. 3.**
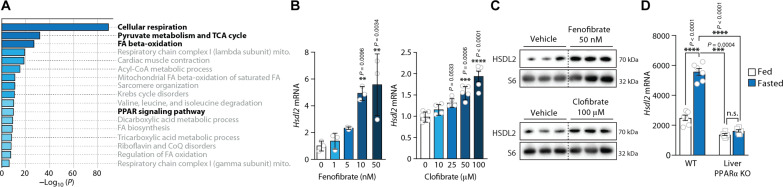
The expression of *Hsdl2* is induced by PPARα. (**A**) Gene ontology analysis performed with Metascape on the 159 unique genes coexpressed with *Hsdl2* in mouse tissues. A correlation coefficient equal or above 0.7 was fixed to select these genes, which are listed in table S5. Details about the Metascape results are presented in table S6. (**B**) FAO cells were treated for 6 hours with the indicated doses of fenofibrate or clofibrate. RNA was extracted, and *Hsdl2* expression was quantified by RT-qPCR (*n* = 3 per dose of fenofibrate; *n* = 4 to 5 per dose of clofibrate). (**C**) Protein lysates were prepared from the experiment described in (B), and Western blots were performed for the indicated proteins (*n* = 3 per group). (**D**) The publicly available National Center for Biotechnology Information’s Gene Expression Omnibus dataset GSE96559 was used for this analysis (*41*). Briefly, wild-type (WT) and liver-specific PPARα knockout (KO) mice (males and 8 weeks old) were fed ad libitum or fasted for 24 hours (*n* = 6 per genotype per group). Liver samples were collected from each mouse, and microarray analyses were performed. In all panels, data are presented as means ± SEM. In (B), significance was determined by one-way ANOVA with Tukey’s multiple-comparisons test. In (B), only the significant effects versus dose 0 are presented. In (D), significance was determined by two-way ANOVA with Tukey’s multiple-comparisons test. n.s., not significant.

To gain insights into the biological functions of HSDL2, we next looked at its tissue distribution in mice. Here, we took advantage of BioGPS, an online resource that hosts a reference plugin that displays gene expression patterns of many mouse tissues and cell types (*37*, *38*). Using this tool, we observed that the expression of *Hsdl2* was elevated not only in the liver but also in other mitochondria-rich tissues such as the heart, brown adipose tissue, and the kidney (fig. S3A, top). A similar distribution profile was found when we measured *Hsdl2* transcript and HSDL2 protein levels in an independent, in-house tissue library (fig. S3, B and C). To identify the core of genes coexpressed with *Hsdl2* that may inform us on its catabolic functions, we next listed the genes correlating with *Hsdl2* expression in tissues. As presented in table S5, a total of 159 unique genes showed a correlation coefficient with *Hsdl2* equal or above 0.7. Gene ontology analyses of this gene set revealed a notable enrichment for mitochondrial processes including “Cellular respiration,” “TCA cycle,” and “Fatty acid β-oxidation” (Fig. 3A and table S6), further supporting a possible functional link between HSDL2 and the mitochondria.

These gene ontology analyses performed on the genes correlating with *Hsdl2* also revealed a significant enrichment for “Peroxisome proliferator-activated receptor (PPAR) signaling pathway” (Fig. 3A). This observation is worth noting, considering that PPARα is a nuclear receptor known to control the expression of key catabolic genes in various oxidative tissues, including the liver (*39*). Other pathways such as “Cardiac muscle contraction,” “Acyl-CoA metabolic processes,” “Mitochondrial fatty acid beta-oxidation,” and “Regulation of fatty acid oxidation” are also known to be linked to PPARα activation. Here, we found that the expression of *Ppara* and *Hsdl2* showed a similar profile between tissues (fig. S3A, top versus bottom) and measured a significant correlation between these genes in the liver of mice upon fasting (fig. S3D). Analyses of quality-controlled mouse chromatin immunoprecipitation sequencing experiments using the ReMap resource (*40*) revealed that PPARα and PGC1α both interact with the proximal promoter of *Hsdl2* in various experimental contexts (fig. S3E). To demonstrate a role for PPARα in the control of *Hsdl2* expression, we next treated FAO cells with increasing doses of the PPARα agonists clofibrate, fenofibrate, or WY14643. Notably, we found a significant increase in the expression of *Hsdl2* transcript and HSDL2 protein in response to all these agonists (Fig. 3, B and C, and fig. S3, F and G). A rise in *Hsdl2* expression was also observed in primary hepatocytes exposed to a PPARα agonist (fig. S3H).

To define whether the *Hsdl2* expression upon fasting is under the dependence of PPARα, we analyzed a publicly available National Center for Biotechnology Information’s Gene Expression Omnibus dataset in which transcriptomics analyses were performed in the liver of PPARα knockout mice (GSE96559) (*41*). As shown in Fig. 3D, we observed that the rise in *Hsdl2* expression upon fasting was completely blunted in mice lacking PPARα. In this experiment, *Hsdl2* was regulated as other classical PPARα target genes, including hydroxyacyl-CoA dehydrogenase trifunctional multienzyme complex subunit alpha (*Hadha*) and medium-chain specific acyl-CoA dehydrogenase, mitochondrial (*Acadm*; fig. S3I). Because PGC1α acts as a key coactivator driving PPARα activity (*42*), we next tested its role in regulating *Hsdl2* expression in vivo. Mice were injected with adenoviruses to overexpress PGC1α in the liver, and the expression of *Hsdl2* was assessed. Notably, we observed a significant rise in hepatic *Hsdl2* levels upon PGC1α overexpression (fig. S3J). Together, these results indicate that HSDL2 is a mitochondrial protein whose expression is driven by the PGC1α-PPARα axis in catabolic hepatocytes.

### The expression of *Hsdl2* is repressed by insulin downstream of mTORC1

**Fig. 4.**
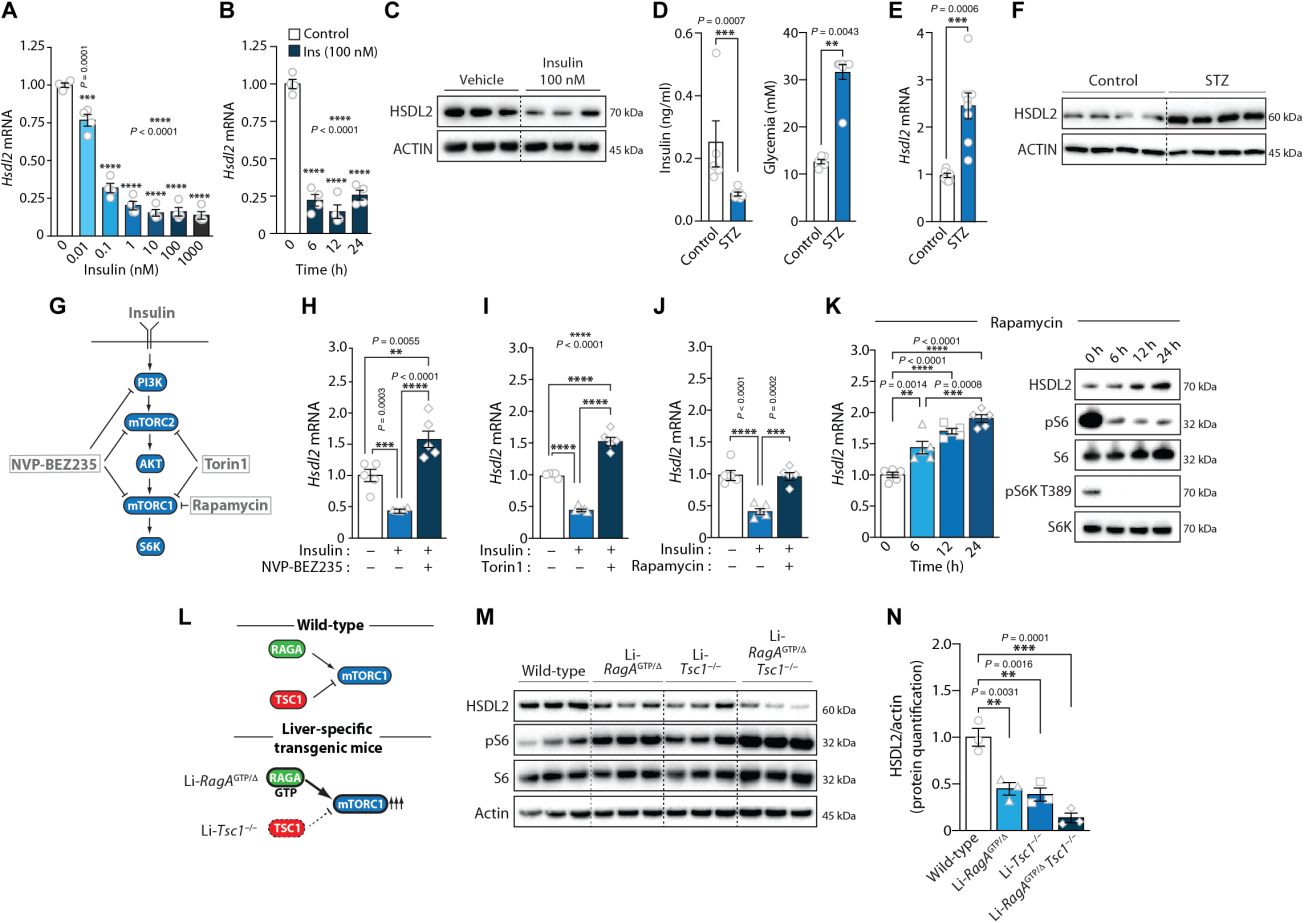
The expression of *Hsdl2* is repressed by insulin downstream of mTORC1. (**A**) FAO cells were serum-deprived for 12 hours and exposed to the indicated doses of insulin for 12 hours. RT-qPCR was performed after treatment (*n* = 4 per condition). (**B**) FAO cells were serum-deprived for 12 hours and were next treated with insulin for the indicated times. RT-qPCR was performed (*n* = 4 per condition). (**C**) FAO cells were serum-deprived for 12 hours and next treated with insulin for 12 hours. Proteins were extracted, and Western blots were performed. (**D**) Mice were injected with saline (*n* = 6) or STZ (*n* = 8). Seven days after injection, blood glucose and insulin were measured. (**E**) The mice used in (D) were euthanized, and liver samples were collected. RT-qPCR was performed. (**F**) Liver proteins were extracted from mice described in (D), and Western blots were performed. (**G**) Simplified overview of the insulin signaling pathway. The inhibitors used in this study are presented. (**H** to **J**) FAO cells were serum-starved for 12 hours. A group of cells was next treated with insulin (10 nM) with or without a pretreatment of 6 hours with (H) NVP-BEZ235, (I) Torin1, or (J) rapamycin. RT-qPCR was performed (*n* = 5 per condition). (**K**) FAO cells were treated with rapamycin for the indicated times. Left: *Hsdl2* mRNA expression was measured by RT-qPCR (*n* = 5 per condition). Right: Proteins were extracted, and Western blots were performed. (**L**) Presentation of the genetic models used to study the impact of hyperactive mTORC1 signaling in mouse liver. (**M**) Mice were fasted for 24 hours. Liver proteins were extracted, and Western blots were performed. (**N**) HSDL2 protein quantification in the experiment presented in (M). In all panels, data are presented as means ± SEM. In (D) and (E), significance was determined by two-tailed, unpaired *t* test. In (A), (B), (H) to (K), and (N), significance was determined by one-way ANOVA with Tukey’s multiple-comparisons test.

The transition between anabolism and catabolism in response to feeding and fasting cycles is highly dependent on circulating insulin levels. Upon feeding, the rise in insulinemia drives anabolism in hepatocytes by turning on the activity of several effectors, including PI3K, mTORC1, and mTORC2 (*43*, *44*). Studies also show that insulin blocks catabolism in the liver, at least in part by repressing PPARα (*45*, *46*). Because *Hsdl2* levels are elevated in catabolic hepatocytes and because its expression increased upon fasting, we first tested whether blocking PI3K-mTOR signaling with serum depletion could induce *Hsdl2* levels. A significant rise in *Hsdl2* expression was measured in response to serum removal (fig. S4A). We next tested whether insulin could repress *Hsdl2* expression in hepatocytes. Serum-deprived FAO cells were exposed to different concentrations of insulin, and *Hsdl2* expression was assessed. As depicted in Fig. 4A, insulin dose-dependently repressed *Hsdl2* levels. This effect occurred within 6 hours following insulin treatment (Fig. 4B). HSDL2 protein levels were also repressed by insulin (Fig. 4C). To define the importance of insulin in controlling hepatic *Hsdl2* expression in vivo, we performed several experiments in mice. First, mice were fasted for 24 hours and then refed for 2 hours to promote insulin secretion. Supporting the results produced in vitro, we found that refeeding significantly reduced *Hsdl2* expression in mouse liver (fig. S4B). In complementary experiments, mice were injected with streptozotocin (STZ), an alkylating agent that disrupts β cell in the pancreas, impairs insulin production, and induces type 1 diabetes in mice. As expected, STZ-injected mice had low circulating insulin and were hyperglycemic (Fig. 4D). Supporting a role of insulin in regulating *Hsdl2* expression, we measured a significant increase in *Hsdl2* transcript and HSDL2 protein in the liver of mice injected with STZ (Fig. 4, E and F). Other classic genes regulated by insulin, including phosphoenolpyruvate carboxykinase (*Pepck*) and *Pgc1a*, were also increased in STZ-injected mice (fig. S4C). These results indicate that insulin represses HSDL2 both in vivo and in vitro.

We next designed experiments to define how insulin controls *Hsdl2* expression. Cells were serum-deprived and exposed to insulin, with or without pharmacological inhibitors of key effectors of the insulin signaling pathway (Fig. 4G). We found that insulin-mediated repression of *Hsdl2* was blocked by NVP-BEZ235, a broad PI3K/mTOR inhibitor (Fig. 4H). Blocking both mTORC1 and mTORC2 with Torin1 also prevented the effect of insulin on *Hsdl2* expression (Fig. 4I). The selective inhibition of mTORC1 with rapamycin led to the same effect, indicating a key role for this kinase in regulating *Hsdl2* levels (Fig. 4J). Consistent with these results, rapamycin time-dependently increased *Hsdl2* mRNA and HSDL2 protein in these cells (Fig. 4K). The same effect was observed in response to NVP-BEZ235 and Torin1 (fig. S4, D and E). Together, these finding show that insulin represses *Hsdl2* expression by activating the mTORC1 signaling pathway.

To confirm the importance of mTORC1 in regulating HSDL2 levels in vivo, we took advantage of established transgenic mouse models with liver-specific mTORC1 hyperactivation (*45*, *47*). These models include mice expressing a constitutively active form of *RagA* in the liver (Li-*RagA*^GTP/Δ^), mice with liver-specific deletion of tuberous-sclerosis complex 1 (Li-*Tsc1*^−/−^), and double transgenic mice with both genes targeted (Li-*RagA*^GTP/Δ^ *Tsc1*^−/−^; Fig. 4L). Control and transgenic mice were fasted for 24 hours and euthanized. As expected, elevated phosphorylation of S6, a downstream effector of mTORC1, was observed in the liver of Li-*RagA*^GTP/Δ^ and Li-*Tsc1*^−/−^, an effect that was even stronger in the liver of Li-*RagA*^GTP/Δ^ *Tsc1*^−/−^ mice (Fig. 4M). Supporting the role of mTORC1 in repressing HSDL2, we found a marked reduction in hepatic HSDL2 protein in all these models (Fig. 4, M and N). HSDL2 protein was more robustly decreased in the liver of Li-*RagA*^GTP/Δ^ *Tsc1*^−/−^ mice. We also noticed a decrease in *Hsdl2* mRNA levels in response to mTORC1 hyperactivation (fig. S4F). Overall, these findings confirm the importance of mTORC1 in repressing HSDL2 in vitro and in vivo.

### HSDL2 depletion impairs mitochondrial respiration and metabolism

**Fig. 5.**
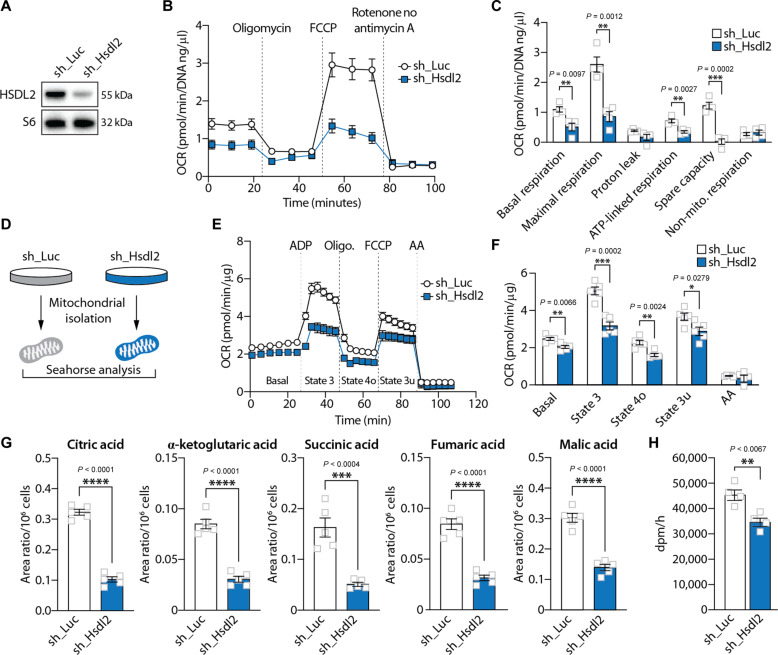
HSDL2 depletion impairs mitochondrial respiration and metabolism. (**A**) HepG2 cells were infected with a control shRNA (sh_Luciferase) or a shRNA targeting HSDL2 (sh_Hsdl2) and then selected with puromycin. Proteins were extracted 7 days after selection, and Western blot was performed. (**B**) OCR measurement in HepG2 cells during the Seahorse Mito Stress protocol (*n* = 5 per condition). (**C**) Respiration parameters calculated from the graph presented in (B). (**D**) HepG2 cells were transduced to express sh_Luciferase or sh_Hsdl2 and then selected with puromycin. Cells were then amplified, and mitochondria were isolated for further experiments. (**E**) OCR measurement in mitochondria extracted from HepG2 cells, following the mitochondria coupling assay protocol (*n* = 5 per condition). (**F**) Respiration parameters calculated form the graph presented in (E). (**G**) Levels of TCA intermediates measured by metabolomics in control and HSDL2-knockdown HepG2 cells (*n* = 5 per condition). (**H**) Measurement of palmitic acid oxidation in control and HSDL2-knockdown HepG2 cells (*n* = 4 per condition). In all panels, data are presented as means ± SEM. In (C) and (F) to (H), significance was determined by two-tailed, unpaired *t* test.

To investigate the functions of HSDL2, human and mouse cell lines (HepG2 and Hepa1-6) were transduced with lentiviruses expressing short-hairpin RNA (shRNA) to knockdown HSDL2. As presented in Fig. 5A and fig. S5A, this approach efficiently reduced HSDL2 protein levels. Because HSDL2 resides in the mitochondria, we first tested the impact of its depletion on cellular respiration. Notably, we observed that HSDL2 knockdown severely reduced oxygen consumption rate (OCR) in both cell lines tested (Fig. 5B and fig. S5B). In detail, a reduction in basal, maximal, and adenosine triphosphate (ATP)–linked respiration was measured in these experiments (Fig. 5C and fig. S5C). We next isolated mitochondria to directly test the impact of HSDL2 depletion on mitochondrial respiration (Fig. 5D). In line with the observations made in cells, we observed a clear reduction in respiration in mitochondria isolated from HSDL2-depleted cells (Fig. 5, E and F). This effect was associated with changes in mitochondrial organization and structure. We found that HSDL2 depletion decreased mitochondrial area, increased mitochondrial circularity, and reduced mitochondrial aspect ratio (fig. S5D), indicating that HSDL2 knockdown reduces mitochondrial branching and increases mitochondrial fragmentation. Metabolomics analyses were next performed to further characterize the impact of HSDL2 on mitochondrial function. As presented in Fig. 5G, we observed a significant reduction in the levels of key TCA cycle intermediates upon HSDL2 depletion, including citric acid, α-ketoglutaric acid, succinic acid, fumaric acid, and malic acid. These results indicate that HSDL2 depletion impairs respiration and TCA cycle activation.

Because HSDL2 expression is increased in response to fasting, a condition linked to a rise in fatty acid oxidation in hepatocytes, we next tested whether the impairment in mitochondrial function observed in HSDL2-depleted cells translated into a defect in fatty acid oxidation rate. Cells were incubated with a radiolabeled fatty acid, and soluble degradation products were isolated and measured. Consistent with the results presented above, a significant reduction in palmitate oxidation was observed in response to HSDL2 knockdown in both human and mouse cells (Fig. 5H and fig. S5E). The same observation was made when HSDL2 was deleted from HEK293 cells using CRISPR-Cas9 (fig. S5, F and G). Overall, these results confirm that HSDL2 depletion impairs fatty acid oxidation.

### HSDL2 controls cholesterol conversion to BAs and FXR activation

**Fig. 6.**
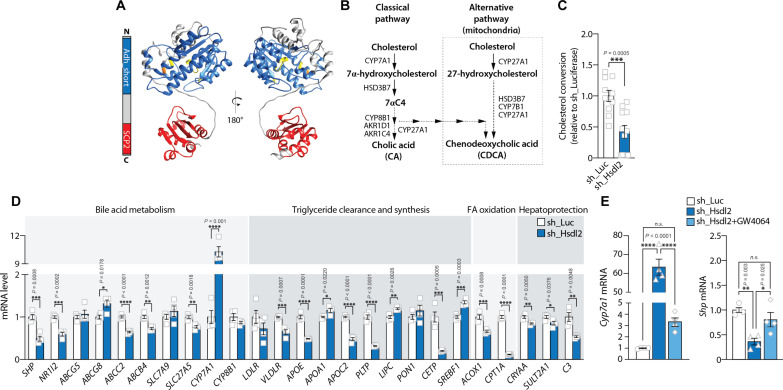
HSDL2 controls cholesterol conversion to BAs and FXR activation. (**A**) HSDL2 structure modeled with AlphaFold. The ADH domain and SCP2 domains of HSDL2 are represented in blue and red, respectively. (**B**) Schematic description of the BA synthesis pathway in hepatocytes. (**C**) Levels of cholesterol conversion to BAs in control and HSDL2-depleted cells. HepG2 cells were exposed to radioactive cholesterol for 24 hours, and cholesterol derivates were extracted using Folch extraction. (**D**) HepG2 cells were infected, selected, and amplified. RNA was extracted, and the expression of genes involved in BA metabolism, triglyceride clearance and synthesis, fatty acid oxidation, and hepatoprotection was measured by RT-qPCR (*n* = 4 per condition). (**E**) HepG2 cells were exposed to vehicle (dimethyl sulfoxide) or GW4064 (20 μM) for 24 hours. Gene expression was then measured by RT-qPCR (*n* = 4 per condition). In all panels, data are presented as means ± SEM. In (C) and (D), significance was determined by two-tailed, unpaired *t* test. In (E), significance was determined by one-way ANOVA with Tukey’s multiple-comparisons test.

SDR are involved in the metabolism of a large variety of compounds, including steroids, prostaglandins, retinoids, lipids, and xenobiotics (*32*). From a structural perspective, SDR family members often display a conserved N-terminal catalytic domain and a highly variable C-terminal substrate binding site. Prediction of HSDL2 structure with AlphaFold showed that the N-terminal region of HSDL2 contains a common α/β-folding pattern characterized by a central β sheet typical of a Rossmann-fold with helices on either side (Fig. 6A and fig. S6A). In this region, a highly conserved NADP^+^ binding site and a catalytic triad are present, which are key functional components of SDR proteins (fig. S6B). In the C-terminal region, HSDL2 contains an SCP2 domain, a structure previously reported to bind lipids, including sterols. Differential scanning fluorescence assays revealed that purified HSDL2 binds cholesterol derivates in vitro (*48*), raising the possibility that HSDL2 might participate in cholesterol conversion in response to metabolic challenges.

BAs are essential for the absorption of dietary fats, and they also play important roles in the regulation of systemic cholesterol, lipid, and glucose homeostasis (*49*). Primary BAs, namely, cholic acid and chenodeoxycholic acid, are produced in hepatocytes from the conversion of cholesterol through a complex series of enzymatic reactions, some of which taking place in the mitochondria (Fig. 6B) (*50*). To test whether HSDL2 plays roles in cholesterol conversion, HepG2 cells were incubated with radiolabeled [^3^H]-cholesterol, and BAs were extracted and counted. As presented in Fig. 6C, HSDL2 depletion significantly reduced cholesterol conversion by cells. The decrease in BA production was not caused by defects in cholesterol import to the mitochondria or reduced mRNA expression of key enzymes regulating BA synthesis (fig. S6, C to E). We rather observed elevations in steroidogenic acute regulatory protein (*STAR*) and cytochrome P450 27 (*CYP27A1*) expression in HSDL2-depleted cells. Together, these results indicate that HSDL2 plays a direct role in promoting mitochondrial conversion of cholesterol and BA production.

BAs bind and activate FXR, a nuclear receptor that controls systemic lipid and glucose homeostasis (*51*–*53*). Activation of FXR leads to the down-regulation of genes involved in lipogenesis and the up-regulation of genes involved in fatty acid oxidation and triglyceride clearance. FXR is also involved in the feedback regulation of BA synthesis (*49*). To define whether the defect in BA production observed in HSDL2-depleted cells was associated with changes in FXR activity, we measured the expression of 25 genes previously reported to be regulated by this nuclear receptor (*54*–*57*). As shown in Fig. 6D, this analysis showed that a majority of FXR target genes were affected by HSDL2 knockdown. Notably, the directionality of these changes clearly pointed to a defect in FXR activation in HSDL2-depleted cells (fig. S6F). In these experiments, cytochrome P450 7A1 (*CYP7A1*) was the gene whose expression was most affected by HSDL2 depletion (Fig. 6D). *CYP7A1* is highly sensitive to FXR inhibition and participates in the feedback regulation of BA production. FXR indirectly controls *CYP7A1* levels by promoting the expression of small heterodimer partner (*SHP*), a potent repressor of *CYP7A1* transcription (fig. S6G) (*49*). Following this model, we observed that HSDL2 depletion reduced *SHP* expression (Fig. 6D). To define whether the transcriptional changes induced by HSDL2 knockdown could be rescued by stimulating FXR, we next treated HSDL2-depleted cells with the synthetic FXR agonist GW4064. As shown in Fig. 6E, GW4064 treatment normalized *CYP7A1* and *SHP* expression in these cells. These results show that mitochondrial HSDL2 controls BA production and FXR activation in hepatocytes.

As presented in Fig. 5, HSDL2 knockdown was associated with a significant reduction in mitochondrial respiration and fatty acid oxidation. Because the FXR agonist GW4064 could rescue, in part, the transcriptional defects observed in HSDL2-depleted cells (Fig. 6E), we next tested whether reactivating FXR could correct mitochondrial function in the same experimental context. Here, we observed that GW4064 failed to correct respiration in HSDL2-depleted cells (fig. S6, H and I), indicating that acute restoration of FXR signaling is not sufficient to overcome mitochondrial dysfunction in these cells.

### *Hsdl2* expression associates with circulating BA levels in mice

**Fig. 7.**
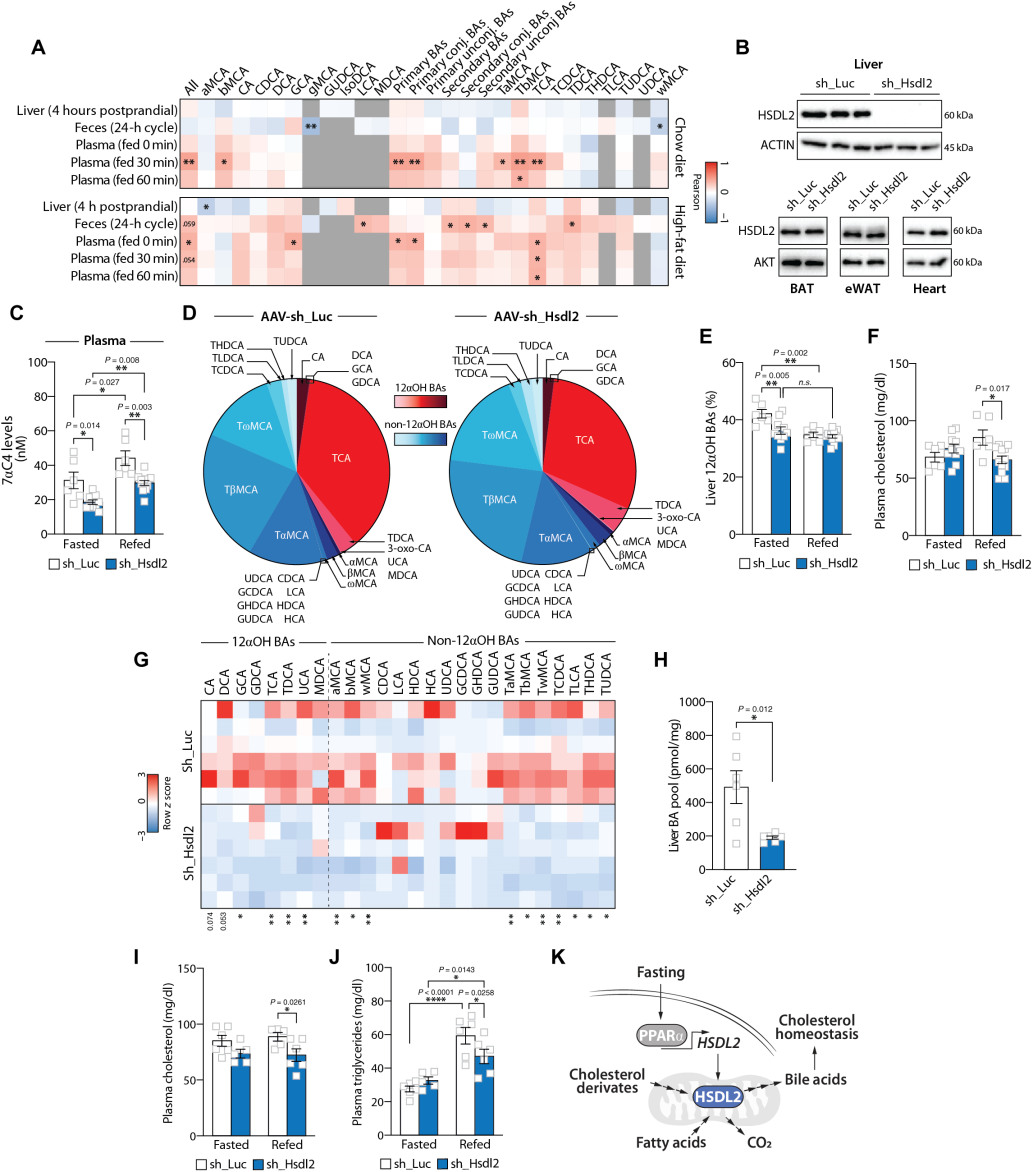
Liver-specific HSDL2 depletion affects hepatic BA metabolism and circulating cholesterol in mice. (**A**) Heatmap showing the association between hepatic *Hsdl2* expression and BA species in the liver, feces, and plasma from chow- or high-fat diet–fed mice. (**B**) Protein lysates were prepared from tissues collected from control and liver-specific HSDL2-knockdown mice, and Western blots were performed. (**C**) Mice were euthanized following either a fasting or a refeeding period (Fasted: sh_Luc, *n* = 6, and sh_Hsdl2, *n* = 10; Refed: sh_Luc, *n* = 6, and sh_Hsdl2, *n* = 12). 7α-Hydroxy-4-cholesten-3-one (7αC4) levels were measured in the plasma. (**D**) Hepatic BA profiling from control and liver-specific HSDL2-knockdown mice after 24 hours of fasting. Values of individual BAs are normalized to total BA pool. (**E**) Proportion of 12αOH BAs in the liver of mice described in (C). (**F**) Plasma cholesterol levels measured in mice described in (C). (**G**) Heatmap showing the liver BA composition of control and liver-specific HSDL2-knockdown mice after a 2-hour refeeding period with a high-cholesterol diet (sh_Luc, *n* = 6; sh_Hsdl2, *n* = 6). Values of BAs were log-transformed to produce the heatmap. (**H**) Total hepatic BA pool of control and liver-specific HSDL2-knockdown mice after a 2-hour refeeding period with a high-cholesterol diet (sh_Luc, *n* = 6; sh_Hsdl2, *n* = 6). (**I**) Plasma cholesterol levels measured in mice described in (G) (Fasted: sh_Luc, *n* = 6, and sh_Hsdl2, *n* = 5; Refed: sh_Luc, *n* = 5, and sh_Hsdl2, *n* = 6). (**J**) Plasma triglyceride levels measured in mice described in (G) (Fasted: sh_Luc, *n* = 6, and sh_Hsdl2, *n* = 5; Refed: sh_Luc, *n* = 6, and sh_Hsdl2, *n* = 6). (**K**) Schematic presentation of the proposed functions of hepatic HSDL2. In all panels, data are presented as means ± SEM. In (C), (E), (F), (I), and (J), significance was determined by two-way ANOVA with Tukey’s multiple-comparisons test. In (H), significance was determined by two-tailed, unpaired *t* test.

To study the physiological link between *Hsdl2* expression and BA metabolism in vivo, we used a powerful multiomics resource recently developed to explore the genetic regulation of BAs and their relevance in health and disease (*58*). In this study, postprandial BAs were profiled in plasma, liver, and feces of 36 genetically diverse mouse strains fed either a chow or a high-fat diet (360 mice in total). Liver transcriptomics and genotyping data were also collected from these mice, thus allowing the identification of physiological modulators of BA metabolism (*58*). Using this resource, we analyzed the relationship between hepatic *Hsdl2* expression and BA levels in various biological compartments. As presented in Fig. 7A, we observed positive associations between *Hsdl2* levels and various circulating BA species. The significant associations were mostly observed 30 min (T30) following a meal test. In detail, total circulating BA pool (*r* = 0.45, *P* = 0.006), primary BAs (*r* = 0.45, *P* = 0.006), primary conjugated BAs (*r* = 0.44, *P* = 0.007), β-muricholic acid (bMCA) (*r* = 0.40, *P* = 0.016), tauro-α-muricholic acid (TbMCA) (*r* = 0.48, *P* = 0.004), TaMCA (*r* = 0.34, *P* = 0.04), and TCA (*r* = 0.44, *P* = 0.007) all correlated with hepatic *Hsdl2* expression in chow-fed mice. The expression of *Hsdl2* and BA levels also positively correlated in high-fat diet–fed mice, although the nature and the dynamics of these associations slightly differed compared to chow-fed animals. Overall, these results support a relation between hepatic *Hsdl2* and postprandial BA homeostasis in vivo.

### Liver-specific HSDL2 depletion alters hepatic BA metabolism and circulating cholesterol in mice

To functionally test the connection between liver HSDL2, BA homeostasis, and systemic metabolism in vivo, mice were injected with adeno-associated virus serotype 2/8 (AAV2/8) expressing shRNA targeting either luciferase or *Hsdl2*. As shown in Fig. 7B, this approach severely repressed HSDL2 levels in the liver but not in any other tissue tested. Mice were next followed for a period of 14 days following AAV2/8 injection. Because HSDL2 expression is dynamically regulated in response to anabolic and catabolic signals, mice were euthanized following either a fasting or a refeeding protocol. An overview of the experimental scheme is presented in fig. S7A. No change in body weight or food intake was measured between the groups (fig. S7, B and C). Liver weight and histological organization were not affected by HSDL2 depletion (fig. S7, D and E).

Because our findings uncovered a potential link between HSDL2 expression and BAs, extended BA profiles were measured by mass spectrometry from liver and plasma of control and liver-specific HSDL2-knockdown mice. Although we were initially expecting a reduction in BA levels upon HSDL2 depletion, we found no clear difference in the levels of individual BA species in the liver, plasma, and feces (tables S7 to S9). The only difference that we found in liver-specific HSDL2-knockdown mice was a decrease in levels of 7α-hydroxy-4-cholesten-3-one, a marker of BA synthesis (Fig. 7C and fig. S7F). This effect was observed in plasma of both fasted and refed mice and in the liver of fasted mice (Fig. 7C and fig. S7F). These observations indicate that hepatic HSDL2 exerts some effects on BA homeostasis in mice, although its depletion in chow-fed mice does not translate into major changes in individual BA species.

Previous studies have shown that conditions such as fasting, PPARα stimulation, and type 1 diabetes, which we found to promote HSDL2 expression (Figs. 1, D and E; 3, B to D; and 4, E and F), often associate with alterations in BA pool composition rather than total BA levels (*59*–*63*). Specifically, an increase in 12α-hydroxylated BAs (12αOH) over non–12α-hydroxylated BAs (non-12αOH) was consistently observed in these conditions. On the basis of these findings, we hypothesized that HSDL2 could play roles in modifying the composition of the BA pool in response to nutritional cues, at least in part by promoting the production of 12αOH BAs. Supporting this model, differences in BA pool composition were found in the liver in response to HSDL2 depletion (Fig. 7D). Precisely, a significant decrease in the percentage of 12αOH BAs was observed in HSDL2-depleted mice upon fasting (Fig. 7E). To define whether the changes in the 12αOH BA proportion observed upon hepatic HSDL2 knockdown was sufficient to affect FXR activity in vivo, we next measured the expression of FXR target genes in the liver of control and liver-specific HSDL2-depleted mice. As shown in fig. S7G, the expression of some FXR targets was reduced upon hepatic HSDL2 knockdown (*Nr1i2*, *Ldlr*, *Apoc2*, *Pltp* and *C3*). Although the effect of HSDL2 knockdown on FXR target genes was less robust in mouse liver compared to human cells in vitro (Fig. 6D), these results support a role for hepatic HSDL2 in regulating BA homeostasis and FXR activation in mice. In a last set of experiment, we measured plasma cholesterol levels to determine whether the modification in BA pool composition linked to HSDL2 depletion could also affect cholesterol homeostasis in vivo. Notably, we observed that hepatic HSDL2 loss significantly reduced cholesterol levels in response to refeeding in mice (Fig. 7F). No difference in circulating cholesterol was found between the groups upon fasting. The impact of HSDL2 depletion on postprandial cholesterol levels were not accompanied by changes in other circulating metabolites, including triglycerides, non-esterified free fatty acid, and glucose (fig. S7, H to J). Together, these results indicate that hepatic HSDL2 depletion induces changes in hepatic BA metabolism, FXR activation, and cholesterol homeostasis in mice.

To test whether HSDL2-depleted mice would exhibit more profound phenotypes if exposed to a different nutritional challenge, control and liver-specific HSDL2-knockdown mice were fed a high-cholesterol diet for a period of 14 days. HSDL2 depletion did not affect food intake and body weight (fig. S7, K and L). Confirming a role for HSDL2 in regulating BA metabolism, we observed significant decreases in the levels of many BA species and a reduction in total hepatic BAs in the liver of HSDL2-depleted mice (Fig. 7, G and H). Specifically, we found that most 12αOH BAs measured were reduced upon HSDL2 depletion. The levels of several non-12αOH BAs were also decreased, indicating that HSDL2 depletion had a deeper impact on hepatic BA profile in mice fed a high-cholesterol diet. Overall, we observed a tendency for a reduction in the proportion of liver 12αOH BAs in the liver of HSDL2-knockdown mice, but this effect did not reach statistical significance (fig. S7M). The changes in BAs induced by hepatic HSDL2 depletion were specifically observed in the liver of mice upon refeeding (table S10). Here, we observed no difference in BA levels in plasma and feces between the groups (tables S11 and S12), indicating that repressing hepatic HSDL2 primarily affected liver BAs in mice fed a high-cholesterol diet. Consistent with observations made in vitro and in vivo, we found a reduction in the expression of some FXR target genes in the liver of HSDL2-depleted mice (fig. S7N). We next measured blood parameters in this study to define the impact of liver HSDL2 depletion on systemic metabolism. As shown in chow-fed animals, we observed a significant decrease in postprandial cholesterol levels in HSDL2-knockdown mice exposed to a high-cholesterol diet (Fig. 7I). We also measured lower circulating triglycerides in these mice (Fig. 7J). Together, these observations support the importance of HSDL2 in regulating BA production and postprandial lipid homeostasis in vivo.

As presented in Fig. 5 and fig. S5, loss of HSDL2 in vitro led to a significant decrease in mitochondrial respiration, an effect associated with a reduction in fatty acid oxidation. To test whether HSDL2 loss similarly affects mitochondrial function in mice, mitochondria were isolated from the liver of control and HSDL2-depleted mice. Following isolation, oxygen consumption was measured in response to pyruvate/malate or palmitoyl-carnitine to assess respiration linked to Complex I and respiration linked to fatty acid β-oxidation, respectively. As presented in fig. S7 (O and P), we found no effect of HSDL2 depletion on Complex I respiration, indicating that repressing HSDL2 does not cause generalized mitochondrial defects in vivo. However, a reduction in β-oxidation was specifically found in mitochondria isolated from HSDL2-depleted mice (fig. S7, Q and R). As described for BA profile and the expression of FXR target genes, the effect of HSDL2 loss on β-oxidation was observed in response to food intake. These results indicate that the impacts of HSDL2 on mitochondrial metabolism are dynamic and influenced by the nutritional status. Overall, our findings position HSDL2 as a factor regulating hepatic and systemic metabolism in response to nutritional cues (Fig. 7K).

## DISCUSSION

Although the understanding of the molecular mechanisms linking nutritional signals to liver metabolism has greatly expanded over the past decades (*64*), the precise nature of the catabolic processes triggered in hepatocytes upon nutritional deprivation remains incompletely characterized. Here, we report the identification of HSDL2 as a protein linking nutritional cues to BA and cholesterol homeostasis. Studies in cell lines and mouse liver revealed that HSDL2 localizes to the mitochondria. We show that the activation of PPARα upon fasting plays an important role in driving the expression of HSDL2. Conversely, the rise in insulin linked to food intake activates the PI3K-mTORC1 axis and represses HSDL2 expression. We report that HSDL2 depletion impairs mitochondrial respiration, fatty acid oxidation, and the activity of the TCA cycle. We found that HSDL2 depletion affects BA metabolism and FXR activation and decreased postprandial cholesterol levels in mice. Our study thus identifies HSDL2 as an emerging regulator of catabolism in hepatocytes.

HSDL2 belongs to the SDR family, a group of proteins that catalyze oxidations and reductions of a wide variety of substrates including steroid hormones, oxysterols, BAs, prostaglandins, retinoids, fatty acids, amino acids, sugars, and xenobiotics (*65*). In humans, more than 70 SDR genes have been identified, but the function of many, including HSDL2, remains to be characterized (*66*). Because SDRs often show activity toward multiple substrates, the identification of their physiological functions is particularly challenging (*67*). Although the identity of the endogenous ligands for HSDL2 is unknown, differential scanning fluorescence assays performed in vitro showed that purified HSDL2 binds NADP/H, sterol derivates, and fatty acids (*48*). These results are consistent with the fact that HSDL2 has a highly conserved NADP/H binding site and an SCP2 domain, a structural feature that binds sterols and other lipids to promote their transport between membranes (*68*). Here, we show that HSDL2 primarily localizes to the mitochondria in hepatocytes and that its depletion affects BA homeostasis in vitro and in vivo. As extensively reviewed before, BA biosynthesis starts from cholesterol and proceeds through a complex sequence of enzymatic steps compartmentalized in cytoplasm, endoplasmic reticulum, mitochondria, and peroxisomes (*69*). The exact sequence of the biosynthetic steps has still not been defined because many intermediates are substituents for the same enzymes. In addition, how BAs and intermediates are transported between different subcellular compartments is still not well known (*69*). On the basis of HSDL2 structural organization and cellular distribution, it is possible that HSDL2 might control BA homeostasis by promoting the conversion of cholesterol intermediates and their transport between mitochondria and other organelles. Here, we show that hepatic HSDL2 depletion reduced BA levels in the liver of mouse fed a high-cholesterol diet. Specifically, we show that most of the 12αOH BAs measured were reduced upon HSDL2 depletion. The levels of several non-12αOH BAs were also decreased. In chow-fed mice, these effects were less pronounced, but we still found a lower proportion of 12αOH BAs in response to HSDL2 inhibition. Interestingly, the limiting enzyme capable of catalyzing the production of 12αOH BAs is cytochrome P450 family 8 subfamily B polypeptide 1 (CYP8B1), a protein that resides in the endoplasmic reticulum. The expression of hepatic CYP8B1 is regulated exactly as HSDL2: It is (i) increased upon fasting (*59*, *60*), (ii) activated by PPARα agonists (*59*, *62*), (iii) repressed by insulin (*63*), and (iv) elevated in type 1 diabetes (*63*). These observations suggest that HSDL2 might work in coordination with CYP8B1 to trigger the production of 12αOH BAs. Whether mitochondrial HSDL2 catalyzes the production and transport of cholesterol intermediates to support the production of 12αOH BAs by CYP8B1 is an intriguing possibility. Additional studies are needed to test this model.

HSDL2 is a protein whose expression is enriched in catabolic hepatocytes. Experiments performed in various models revealed that PPARα and PGC1α, two transcriptional regulators controlling gene expression during fasting, promote the expression of HSDL2. We also found that insulin, a powerful anabolic signal linked to feeding, activates mTORC1 to decrease HSDL2 expression. These results are consistent with previous reports showing the existence of a cross-talk between mTORC1 signaling and PGC1α-PPARα (*45*, *70*). In hepatocytes, mTORC1 was shown to antagonize PPARα to reduce catabolism upon feeding (*45*). Why HSDL2 expression is tightly controlled by nutritional cues is a central question emerging from our study. Gene expression and metabolomic analyses in numerous mouse strains show that hepatic *Hsdl2* expression positively correlates with postprandial excursion of several BA species in mice. Supporting the link between liver HSDL2 and BA homeostasis, we found that HSDL2 knockdown alters liver BA pool composition and size in mice. One interesting alteration in BA profile that we found in the current study is the reduction in 12αOH BAs upon HSDL2 depletion. Several studies have now reported that fasting and insulin, which both affect HSDL2 levels, also affect 12αOH BA metabolism in mice. For instance, it was reported that fasting increases, whereas insulin decreases, the hepatic proportion of 12αOH BAs (*60*, *62*, *63*). Previous studies show that rising 12αOH BAs increases the hydrophobicity of the BA pool and promotes intestinal cholesterol absorption (*63*). We report here that the depletion of hepatic HSDL2 reduced circulating cholesterol and triglyceride levels upon refeeding in mice. On the basis of these observations, we propose that fasting-mediated activation of HSDL2 might serve to alter BA metabolism to maximize lipid absorption when feeding resumes.

In addition to altering cholesterol conversion to BAs, we found that modulation in HSDL2 also affected mitochondrial fatty acid oxidation and respiration in vitro and in vivo. This observation is interesting considering that, in addition to the liver, HSDL2 is highly expressed in mitochondria-rich tissues such as brown fat, heart, and kidney, which are all notoriously known to use substantial amounts of fatty acids for energy or heat production. As discussed above, purified HSDL2 protein binds fatty acids in addition to cholesterol derivates (*48*), thus suggesting that HSDL2 could, in addition to affecting BA homeostasis, participate in the catabolism of fatty acids in the mitochondria. It is important to point out that the phenotypes associated with HSDL2 depletion were generally more severe in immortalized cell lines compared to mouse liver. This is particularly true regarding mitochondrial function and FXR activation, which were severely affected by HSDL2 depletion in vitro but less in vivo. The reasons for these differences are still unknown. It is possible that the incomplete cholesterol breakdown due to HSDL2 depletion may have led to the buildup of cholesterol intermediates in the mitochondria. Numerous studies now show that mitochondrial accumulation of cholesterol and oxysterols impairs mitochondrial function (*71*, *72*). Because there are significant differences in the catabolic intermediates produced from cholesterol degradation between immortalized and normal hepatocytes (*73*–*75*), differences in the production and recycling of cholesterol derivates and FXR ligands could have contributed to the alteration of mitochondrial function and FXR activation in these experiments. As reported before, important differences exist in the ability of different BAs to bind and activate FXR (*51*, *52*). Last, we cannot exclude the possibility that compensatory mechanisms might have been triggered in mouse liver to palliate the absence of HSDL2 and limit the consequence of its inactivation.

Recent studies have shown that HSDL2 levels are high in several human cancers including brain (*21*), ovarian (*22*), breast (*23*), bladder (*24*), thyroid (*25*), lung (*26*), pancreas (*27*), and cervix (*28*–*30*). In these reports, elevated HSDL2 was often linked to tumor grade and poor overall survival. It was shown that HSDL2 depletion reduces cell proliferation, colony formation, and cell motility, while promoting cell cycle arrest and apoptosis. The mechanism linking HSDL2 to cancer cell survival was not clearly demonstrated in these reports. In some cases, a reduction in protein kinase B/Akt (Akt) expression was observed in response to HSDL2 depletion (*25*, *26*). Lower expression of lipogenic regulators and reduced triglyceride accumulation were also measured in some cancer cell lines upon HSDL2 knockdown (*27*, *28*). However, whether these effects are functionally linked to HSDL2 or are an indirect consequence of impaired cell viability remains to be determined. It is important to point out that we found no proliferative defects upon HSDL2 depletion or deletion in any of the cell lines tested. Consistently, HSDL2 knockdown in mice did not affect hepatocyte viability and apoptosis. The reason for these differences is unclear. It is possible that some cancer cell lines might have developed a dependency to HSDL2, rendering them particularly sensitive to its loss. In addition, we cannot exclude the possibility that HSDL2 might have distinct functions in different cell types. Additional work is needed to better understand the physiological and pathological roles of HSDL2 in different cellular contexts.

In conclusion, we provide evidence that HSDL2 is a fasting-induced mitochondrial protein that links nutritional signals to BA and cholesterol homeostasis. Our study positions hepatic HSDL2 as an important metabolic regulator affecting systemic adaptation to nutritional cues.

## METHODS

### Cell culture and reagents

All the cell lines (HepG2, Hepa 1-6, FAO, and HEK293) were obtained from the American Type Culture Collection (ATCC) and cultured according to standard mammalian tissue culture protocols and sterile technique. Hepa 1-6 and HEK293 cell lines were cultured in complete Dulbecco’s modified Eagle’s medium (DMEM; Wisent, #319-015-CL) supplemented with fetal bovine serum (FBS; 10%; Wisent, #080-150) and penicillin-streptomycin (1%; Wisent, #450-201-EL). HepG2 cells were cultured with low-glucose DMEM (Wisent, #319-010-CL), and FAO cells were cultured with RPMI 1640 (Wisent, #350-000-CL), also supplemented with 10% FBS and 1% penicillin-streptomycin. The following reagents were used in cell culture experiments: rapamycin (LC Laboratories, #R5000), Torin1 (Cayman Chemical, #10997), puromycin (Sigma-Aldrich, #P8833), Blasticidin S-HCl (Sigma-Aldrich, #15205), clofibrate (Cayman Chemical, #10956), fenofibrate (Sigma-Aldrich, #F6020), WY14643 (Cayman Chemical, #70730), NVP-BEZ235 (Cayman Chemical, #10565), insulin (human; Sigma-Aldrich, #I2643), and GW4064 (Cayman Chemical, #10006611). For all in vitro experiments described in Fig. 4 and fig S4, NVP-BEZ235 was used at a concentration of 250 nM, rapamycin was used at a concentration of 100 nM, and Torin1 was used at a concentration of 250 nM.

### Primary hepatocytes

Isolation of primary hepatocytes was adapted from a method previously described (*76*). Briefly, livers from 7- to 10-week-old C57BL/6 male mice were perfused and digested using liver perfusion medium (Gibco, #17701038) and liver digestion medium (Gibco, #17703034), respectively. Livers were then collected, disassembled, filtered, and lastly centrifuged with Percoll to only recover the hepatocytes. Cells were plated at a confluency of 300,000 cells per well with attachment medium [M199 medium (Wisent, #316-025-CL), 10% FBS (Wisent, #080-150), 100 nM T3 (Sigma-Aldrich, #T6397), 500 nM dexamethasone (Sigma-Aldrich, #D4902), 1 nM insulin (Sigma-Aldrich, I2643), and 1% penicillin-streptomycin (Wisent, #450-201-EL)]. Six hours after plating, the attachment medium was replaced by another medium consisting of low-glucose DMEM (Wisent, #319-010-CL), 1× GlutaMAX (Gibco, 35050061), 100 nM dexamethasone (Sigma-Aldrich, #D4902), and 1% penicillin-streptomycin (Wisent, #450-201-EL). This medium was also used for subsequent treatments.

### Virus production and infection

Retroviruses were produced using gag/pol and cytomegalovirus vesicular stomatitis virus glycoprotein as the packaging system. Lentiviruses were produced using psPAX2 and pMD2G. 293T cells were transfected with the vectors. Virus-containing supernatants were collected at 48 hours after transfection and filtered using a 0.45-μm filter. Cells were transduced for 24 hours in the presence of polybrene (8 μg/ml). After infection, the cells were dispersed into fresh medium. Cells were selected on the following days with either puromycin or blasticidin, depending on the viral constructs. In detail, HepG2 cells were selected with puromycin (5 μg/ml) or blasticidin (20 μg/ml); Hepa 1-6 cells were selected with puromycin (4 μg/l) or blasticidin (4 μg/ml), and HEK293 cells were selected with puromycin (2 μg/ml).

### Vectors

Lentiviral shRNAs were obtained from the collection of The RNAi Consortium (TRC) at the Broad Institute. These shRNAs are named with the numbers found at the TRC public website: sh_Luciferase (TRCN0000072254), sh_Hsdl2 human (TRCN0000064933), sh_Hsdl2-1 (TRCN0000099635), and sh_Hsdl2-2 (TRCN0000376104). Single guide RNA (sgRNA) guides targeting human HSDL2 were inserted in the pX459 V2.0 plasmid [pSpCas9(BB)-2A-Puro, Addgene #62988] to knockout HSDL2 in HEK293 cells. The sgRNA sequences were TTTATCACAGGTGCAAGCCGTGG (43fw) and ACAGGTGCAAGCCGTGGCATTGG (49fw). These guides specifically targeted the exon 2 of *Hsdl2*.

### Glucose production assay

The Amplex Red Glucose/Glucose Oxidase Kit (Invitrogen, A22189) was used to measure glucose production in FAO clonal cell lines. Briefly, FAO clones were plated at a concentration of 125,000 cells per well in 12-well plates. The next day, cells were serum-deprived and treated for 5 hours with hepatic glucose production medium [DMEM without glucose (Sigma-Aldrich, S5761), 44 mM sodium bicarbonate (Sigma-Aldrich, S5761), 2 mM sodium pyruvate (Sigma-Aldrich, P2256), and sodium l-lactate (Sigma-Aldrich, L7022)]. The pH was adjusted to 7.3 with 5 M NaOH. After the treatment, medium was collected to detect glucose production according to the kit instructions. For each clone, several wells were treated with insulin as negative controls, and other wells were lysed with 50 mM NaOH to measure protein content with the Bradford technique. Glucose production was normalized to protein content and presented as fold change compared to the parental FAO cell line.

### Western blotting

Following procedures previously described (*77*), all cells were rinsed twice with room-temperature phosphate-buffered saline (PBS) before lysis. Cells were lysed with Triton-X 100 containing lysis buffer [50 mM Hepes (pH 7.4), 2 mM EDTA, 10 mM sodium pyrophosphate, 10 mM sodium glycerophosphate, 40 mM NaCl, 50 mM NaF, 2 mM sodium orthovanadate, 1% Triton-X 100, and one tablet of EDTA-free protease inhibitors per 25 ml]. Tissues were homogenized with the same buffer supplemented with 0.1% sodium lauryl sulfate and 1% sodium deoxycholate. Cells and tissues were rotated at 4°C for 10 min, and then, the soluble fractions of cell lysates were isolated by centrifugation for 10 min in a microcentrifuge. Protein levels were then quantified using Bradford reagent and analyzed by Western blotting. Protein extracts were diluted in sample buffer, denatured by heat (95°C) for 10 min, and loaded on precast gels (Life Technologies). Proteins were transferred to polyvinylidene difluoride membranes blocked in 5% milk diluted in PBS-Tween and incubated with their primary antibody overnight at 4°C. The following antibodies were used: HSDL2 (dilution, 1:20,000; Proteintech, 15631-1-AP), actin (dilution, 1:2000; Cell Signaling Technology, 4967S), pS6 S240/244 (dilution, 1:1000; Cell Signaling Technology, 5364S), S6 (dilution, 1:1000; Cell Signaling Technology, 2217S), pS6K T389 (dilution, 1:1000; Cell Signaling Technology, 9205S), S6K (dilution, 1:1000; Cell Signaling Technology, 9202S), pAKT S473 (dilution, 1:1000; Cell Signaling Technology, 9271S), and pan-AKT (dilution, 1:1000; Cell Signaling Technology, 4691S). Secondary antibodies were purchased from Cell Signaling Technology (dilution, 1:5000; Cell Signaling Technology, #7074 and #7076). ChemiDoc MP Imager and ChemiDoc and Image Lab software (version 6.1) were used to acquire and analyze images.

### Microarray analyses

As previously described (*77*), whole-genome gene expression was performed using the Affymetrix Rat Gene 2.0 ST Array. The RNA was labeled and hybridized using a standard Affymetrix protocol. The quality of arrays was judged using standard quality control parameters, and all arrays passed the quality control filters. Expression values were extracted using the Robust Multichip Average method (*78*, *79*) implemented in the oligo package in R (*80*). Raw data from these analyses can be found at this link doi:10.5061/dryad.rxwdbrvhk.

### Quantitative real-time polymerase chain reaction

Total mRNA was isolated from cells and tissues using the Monarch Total RNA Miniprep Kit (New England Biolabs, T2010S). RNA concentration was estimated from absorbance at 260 nm. cDNA synthesis was performed using the iScript Advanced cDNA Synthesis Kit for quantitative real-time polymerase chain reaction (RT-qPCR; Bio-Rad). mRNA extraction and cDNA synthesis were performed following the manufacturer’s instructions. cDNA was diluted in deoxyribonuclease-free water (1:15) before quantification by real-time PCR. mRNA transcript levels were measured in duplicate samples using CFX96 or CFX384 touch real-time PCR (Bio-Rad, Mississauga, ON, Canada). Chemical detection of the PCR products was achieved with SYBR Green (Bio-Rad, 172-5274). At the end of each run, melt curve analyses were performed, and representative samples of each experimental group were run on agarose gel to ensure the specificity of amplification. Gene expression was corrected for the expression level of the reference gene (*77*). The primer sequences used are presented in table S13.

### Sequence conservation analysis

The analysis of HSDL2 conservation between species was performed using the Constraint-based Multiple Alignment Tool, a multiple-sequence alignment tool that finds a collection of pairwise constraints derived from conserved domain database, protein motif database, and sequence similarity, using RPS-BLAST, BLASTP, and PHI-BLAST (*81*).

### Mitochondria isolation

Mitochondria were isolated using different protocols. For the experiments presented in Fig. 2 (D and E), mitochondria were isolated using the Mitochondria Isolation Kit for Cultured Cells from Abcam (ab110171) according to the manufacturer’s instructions. Briefly, cells were collected and disrupted in a 2-ml Dounce homogenizer. A first centrifugation was performed at 4°C for 10 min, and the supernatant was saved. A second disruption and a centrifugation step were performed, and the two supernatants were combined. A last centrifugation was performed to collect the mitochondrial pellet. This pellet was resuspended in the appropriate buffer supplemented with protease inhibitors. For the experiments described in Fig. 5E, cells were harvested using a cell scraper in PBS and resuspended in mitochondria isolation buffer: 300 mM sucrose, 1 mM EGTA/tris, 5 mM Mops/tris, 5 mM KH_2_PO_4_, adjusted to pH 7.4 with KOH. The suspension was then homogenized using a Teflon pestle in a glass tube and filtered through 40-μm, 10-μm, and 5-μm polyethylene terephthalate filters (pluriSelect, Germany). The filtrate was centrifuged at 6000*g* for 10 min, and the supernatant was discarded and replaced with fresh isolation buffer. Protein content of the mitochondrial extraction was measured using the protein assay dye (Bio-Rad, USA) based on the Bradford method according to the manufacturer’s protocol. For the isolation of mitochondria from mouse liver, one liver lobe was collected and homogenized in Potter-Elvehjem homogenizer containing 10 ml of isolation buffer [70 mM sucrose, 220 mM mannitol, 10 mM Hepes, and 1 mM EDTA (pH 7.4)]. The homogenate was centrifuged at 900*g* for 10 min at 4°C, and the supernatant was collected and separated in 1.5-ml tubes. The tubes were centrifuged at 10,000*g* for 10 min to isolate mitochondria. The pellet of isolated mitochondria was washed two times in 1 ml of isolation buffer supplemented with 0.5% bovine serum albumin (BSA)–free fatty acid, at 10,000*g* and 4°C for 7 min. Mitochondrial pellets were washed one more time with isolation buffer (10,000*g*, 10 min, and 4°C) to remove BSA. Last, mitochondrial pellets were resuspended in 150 μl of isolation buffer, and protein concentration was assayed using a BCA Protein Assay Kit (23225, Thermo Fisher Scientific).

### Oxygen consumption

HepG2 cells were seeded in a Seahorse 24-well plate at a density of 25,000 cells per well. Twenty-four hours later, cells were washed three times with Seahorse XF medium [DMEM without phenol red supplemented with pyruvate (1 mM), glutamine (4 mM), and glucose (5.5 mM)], and the Seahorse Mito Stress protocol was run. Oxygen consumption was assessed by the sequential addition of oligomycin (1.5 μM), carbonyl cyanide *p*-trifluoromethoxyphenylhydrazone (FCCP; 2 μM), and a mix of rotenone (1 μM) and antimycin A (1 μM). The OCR measurements were normalized to total DNA content, and parameters were calculated using the Seahorse Wave Desktop software from Agilent. For the mitochondrial coupled assay, mitochondria from HepG2 cells were isolated and seeded in a Seahorse 24 well plate at a concentration of 5 mg/ml in 50 μl of mitochondrial assay solution (MAS; 70 mM sucrose, 220 mM mannitol, 5 mM KH_2_PO_4_, 5 mM MgCl_2_, 2 mM Hepes, 1 mM EGTA, and 0.2% free-fatty acid BSA). The plate was centrifuged at 4°C for 10 min at 2000*g* before adding 450 μl of MAS supplemented with 5.5 mM succinate per well. The plate was then incubated for 10 min at 37°C, and the protocol on Wave was run. Oxygen consumption was assessed by sequential addition of adenosine diphosphate (ADP; 4 mM), oligomycin (2 μM), FCCP (4 μM), and antimycin A (4 μM). The parameters were calculated using the Seahorse Wave Desktop software from Agilent. Respiratory measurements of cytosolic mitochondria isolated from mouse liver were performed as described previously (*82*). Briefly, 5 μg of mitochondrial suspension was seeded onto a Seahorse XFe96 plate (Agilent, 103792-100) to measure the pyruvate+malate-driven respiration (complex I) and 4 μg for the palmitoyl-carnitine–driven respiration (β-oxidation). The plate was centrifuged at 2000*g* for 5 min at 4°C, brake off, and an additional 130 μl of respiration buffer was added to each well. The final substrate concentration in the respiration buffer was as follows: pyruvate 5 mM + 5 mM malate and 2 mM malate + 80 μM palmitoyl-carnitine (injected with ADP). The plate was incubated for 5 min at 37°C without CO_2_. OCR was then measured for each substrate in response to the sequential injection of ADP (2 mM), with substrates to induce state 3, oligomycin (3 μM) to induce state 4o, FCCP (4 μM) to determine maximal respiration, and antimycin A (4 μM) to block respiration. The OCR measurements were normalized to protein content.

### Mitochondrial morphology assessment

Mitochondrial morphology was assessed in HepG2 cells, using MitoTracker deep red staining and live-cell imaging. The mitochondria were imaged using Leica SP8 confocal microscope with 63× oil objective, and image analysis was done using the Fiji/ImageJ software. The mitochondria segmentation and quantification were done with the help of the trainable weka segmentation plugin, a machine-learning tool for pixel classification (*83*, *84*). First, the input pixels were initially labeled into two classes, mitochondria and background, and used as a training set for the classifier. To improve the accuracy of the classifier, different training features such as Gaussian blur, Sobel, Gaussian difference, Hessian, and membrane detectors were used to reduce noise, identify object boundaries and corners, and locate membranous structures. Then, a fast random forest algorithm (RF) was applied to generate a classifier based on the training set. After training the RF classifier, every cell image was converted to a binary image containing only mitochondria or a background. The mitochondria objects detected were then evaluated by area expressed in square micrometers, circularity, roundness, and aspect ratio using Fiji’s shape descriptors measurement.

### Immunofluorescence

HepG2 cells were infected with pMXs-3XHA-EGFP-OMP25 and blasticidin-selected. Cells were then plated on poly-lysine–coated slides in 12-well plates. Slides were fixed in 4% paraformaldehyde, blocked, and stained overnight with HSDL2 antibody (1:200) in 5% donkey serum + 0.2% Triton X-100 diluted in PBS. Following the incubation with the primary antibody, slides were washed and incubated with the secondary antibody (1:1000; donkey anti-rabbit 568; Life Technologies, A10042) for 1 hour in the dark at room temperature. After PBS washing, slides were mounted using DAPI-Fluoromount G (Electron Microscopy Sciences) and imaged by confocal microscopy (Microscope LSM800, Zeiss Axio Observer Z1).

### Liver histology

Liver tissue samples were fixed for 48 hours in 10% formalin at 4°C. Tissues were next dehydrated, embedded in paraffin, and cut into 10-mm-thick sections. Sections were stained with hematoxylin and eosin. All pictures were taken on an Olympus BX60 microscope (Tokyo, Japan).

### Liver glycogen extraction and quantification

Liver glycogen content was extracted and measured as described previously (*85*).

### Palmitate oxidation measurement

Palmitic acid [1-14C] was purchased from American Radiolabeled Chemical. We used a protocol adapted by Wanders *et al.* (*86*). Briefly, cells were seeded at a density of 500,000 cells per vial and exposed to 50 μl of labeled palmitic acid at a concentration of 100 μM in Ham’s F10 medium deprived of FBS. Each vial had a perforated cap equipped with a rubber septum and contained a NaOH compartment above the cells. Cells were incubated for 3 hours in a shaking water bath at 37°C. The reaction was terminated by adding 150 μl of 2 M perchloric acid and then left for 3 hours at room temperature to ensure complete trapping of [^14^C]CO_2_ as sodium carbonate in the NaOH present above the cells. Vials were then uncapped, and radioactivity in sodium hydroxide was measured. The acid-soluble products (trapped in the cells) were transferred to tubes containing 90 μl of 20% BSA. The tubes were vortexed and centrifuged at 12,000*g* for 2 min at 4°C to precipitate the denatured proteins. The radioactivity in the supernatant was then quantified. Results are presented as the sum of the radioactivity present in acid-soluble products and the radioactivity trapped in sodium hydroxide.

### Metabolomics for the measurement of TCA cycle intermediates

Cells were first plated at a density of 1,000,000 cells in a 6-cm dish before being processed for gas chromatography–mass spectrometry (GC-MS) analysis as previously described (*87*, *88*). Briefly, 24 hours after plating, cells were rinsed with ice-cold NaCl (0.9%) solution on ice before being scraped with 500 μl of cold 80% methanol on dry ice and stored at −80°C. On the day of analysis, samples were processed for derivatization using a two-step protocol: (i) methoxyamination, using the method described by Fiehn (*89*) and (ii) silylation with MTBSTFA/TBDMCS, using the modified method by Patel *et al.* (*90*). Samples were then analyzed by GC-MS using an Agilent 8890 GC equipped with a DB5-MS + DG capillary column connected to an Agilent 5977B MS operating under electron impact ionization at 70 eV (Agilent Technologies, USA). The Agilent MassHunter Workstation Software was then used for analysis, and after deconvolution, the NIST/EPA/NIH Mass Spectral Library (NIST 2017, Gaithersburg, MD, USA) was used to identify target metabolites. Relative metabolite levels were normalized with the internal standard Myristic acid-d27 (CDN Isotopes, Canada), and data were normalized per million cells.

### Cholesterol conversion into BAs

HepG2 cells were plated at a confluency of 1.5 × 10^6^ cells in a 10-cm dish. Cells grew for 24 hours and were then exposed to 2.5 μCi of C1,2-^3^H(N) cholesterol for 24 hours. Cholesterol conversion was measured using the method of Folch (*91*). Briefly, cells were scraped in 700 μl of PBS and transferred to a glass tube. A total of 100 μl was conserved to dose protein content. After the addition of 3 ml of a chloroform:methanol (2:1) mixture, the tubes were vortexed and centrifuged at 2000*g* for 10 min. The upper phase containing polar molecules (including BAs) was collected to determine the radioactivity content by using a liquid scintillation counter.

### BAs measurement

Plasma, liver, cells, and medium BAs were assessed using liquid chromatography–tandem mass spectrometry (LC-MS/MS) with an electrospray interface, as previously described (*92*). The chromatographic system consisted of an Alliance 2690 HPLC apparatus (Waters), and the MS/MS system was an API3200 mass spectrometer (Applied Biosystems).

### Knockdown of *Hsdl2* in mice

The AAV2/8 plasmids used to knockdown HSDL2 contain an expression cassette consisting of the TBG promoter and U6 promoter, and bGH polyA flanked by AAV2 inverted terminal repeats. The shRNA gene was inserted into the multiple cloning sites between the U6 promoter and AAV2 ITR. Two different shRNA targeting *Hsdl2* were inserted and packaged in AAV2/8: CCCACTGTCATGTAAGGACAT (TRCN0000099635) and TGAACAATGCCAGTGCTATTA (TRCN0000376104). AAV2/8 vectors were packaged by the Canadian Neurophotonics Platform (Centre de recherche CERVO, Québec, Canada). Briefly, viral particles were generated from a triple transfection of HEK293T/17 cells (ATCC, CRL-11268) and collected from the culture media 5 days after transfection. They were concentrated using a tangential flow filtration setup (Vivaflow 50R 100-kDa MWCO, Sartorius) and then purified by iodixanol gradient and ultracentrifugation. Purified particles were collected in suspension buffer (PBS containing 320 mM NaCl, 5% d-sorbitol, and 0.001% Pluronic F-68) and titrated by qPCR (TaqMan) using ITR-based probe and primers. Physical titer and purity were confirmed by separating identical volumes of AAV in 10% SDS–polyacrylamide gel electrophoresis (stain-free; Bio-Rad) in tris-glycine-SDS buffer. Male C57BL/6J mice (10 weeks old; Jackson Lab, strain #000664) were injected with 100 μl of AAV2/8-GFP or AAV2/8-shHSDL2 via the tail vein (1 × 10^11^ plaque-forming units per mouse). The animals were euthanized at the times indicated in the figure legends following the injection.

### Studies with transgenic mice with hyperactive mTORC1 signaling

For liver-specific activation of RagA (Li-*RagA*^*GTP/*Δ^), *RagA^GTP/flox^* mice (*47*) were bred with mice expressing Albumin-Cre (Alb-Cre) recombinase (JAX, stock #003574). For liver-specific deletion of *Tsc1* (Li-*Tsc1^−/−^*), *Tsc1^flox/flox^* mice (JAX, stock #005680) (*93*) were bred with mice expressing Alb-Cre. For the generation of the double transgenic mice carrying both genes targeted (Li-*RagA*^GTP/*Δ*^ *Tsc1*^−/−^), Alb-Cre *RagA^GTP/flox^* mice were crossed with *Tsc1*^*flox/*flox^ mice, as described (*94*).

### Animal care

Studies involving hepatic adenoviral overexpression of PGC1α in mice were approved by the Animal Care Committee of the Institut de recherches cliniques de Montreal (2022-02 JE). Studies with transgenic mice with hyperactive mTORC1 signaling were performed according to protocols approved by the CNIO-ISCIII Ethics Committee for Research and Animal Welfare (CEIyBA) and the Autonomous Community of Madrid (CAM PROEX225.7/22). All the other mouse studies were approved by the Animal Ethics Committee of Université Laval (CPAUL) and performed in accordance with the guidelines of the Canadian Council on Animal Care.

### Statistics

All the statistical analyses were performed using Prism version 9.0.
