# Supplementary material for: HSDL2 links nutritional cues to bile acid and cholesterol homeostasis
Source: Sci Adv. 2024 May 31;10(22):eadk9681. doi: 10.1126/sciadv.adk9681 (PMC11141617; doi:10.1126/sciadv.adk9681)
Supplement: Supplementary file 1 — Figs. S1 to S7 Legends for tables S1 to S13 [file sciadv.adk9681_sm.pdf]

Science Advances
AAAS

Supplementary Materials for

HSDL2 links nutritional cues to bile acid and cholesterol homeostasis

Nolwenn Samson *et al.*

Corresponding author: Mathieu Laplante, mathieu.laplante@criucpq.ulaval.ca

Sci. Adv. **10**, eadk9681 (2024)
DOI: 10.1126/sciadv.adk9681

The PDF file includes:

Figs. S1 to S7
Legends for tables S1 to S13

Other Supplementary Material for this manuscript includes the following:

Tables S1 to S13

Figure S1 - HSDL2 as a protein highly expressed in catabolic hepatocytes.

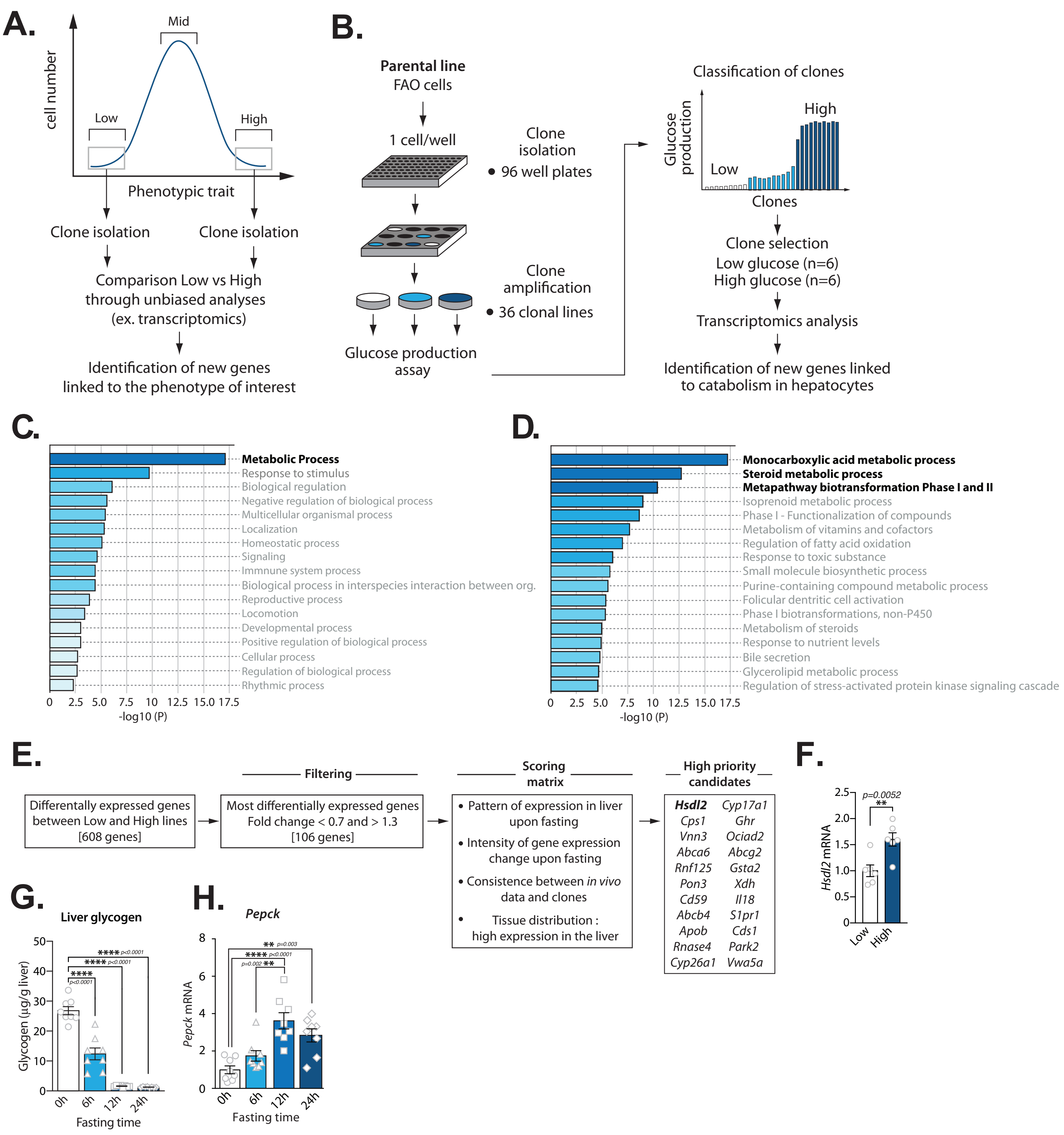

Fig.S1 - HSDL2 is a protein highly expressed in catabolic hepatocytes. (A) Overview of the screening approach for the identification of new genes linked to a phenotype of interest. **(B)** Schematic description of the workflow used to separate Low and High glucose producing clones from FAO parental cell line, leading to the identification of new genes linked to catabolism in hepatocytes. **(C)** Metascape analysis showing the gene ontology parent pathways affected between Low and High lines. **(D)** List of the cellular processes identified in the analysis performed in C. **(E)** Schematic representation of the decision matrix used to prioritize candidate genes linked to catabolism in hepatocytes. **(F)** *Hsdl2* expression measured by RT-qPCR in Low versus High glucose producers isolated as described in B (n=6 per group). **(G)** Male C57BL6J mice were sacrificed after either 0, 6, 12 or 24 hours (n=8/group) of fasting and liver samples were collected. Glycogen was extracted and quantified. **(H)** RNA was extracted from liver samples collected from the experiment described in G and *Pepck* expression was measured by RT-qPCR (n=8/group). In all panels, data are presented as mean ± SEM. In panel F, significance was determined by two tailed, unpaired *t* test. In panel G and H, significance was determined by two-way ANOVA with Tukey's multiple-comparisons test.

Figure S2 - HSDL2 is a mitochondrial protein.

A.

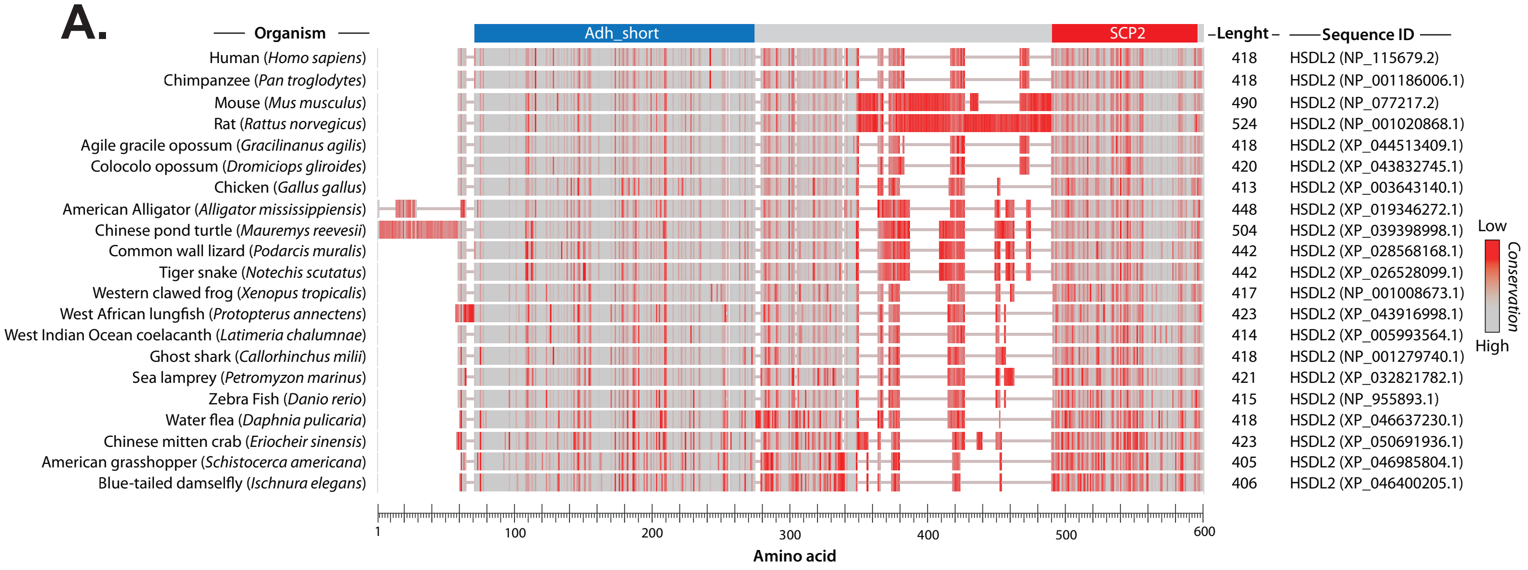

B.

Human HSDL2 [NP_115679.2]
Net charge of query sequence : +2
Analysed region : 58
Number of basic residues in targeting sequence : 7
Number of acidic residues in targeting sequence : 1
Cleavage site : 30
Cleaved sequence : MLPNTGRLAGCTVFITGASRGIGKAIALK
Probability of export to mitochondria: 0.9574

C.

Mouse HSDL2 [NP_077217.2]
Net charge of query sequence : -3
Analysed region : 58
Number of basic residues in targeting sequence : 8
Number of acidic residues in targeting sequence : 1
Cleavage site : 30
Cleaved sequence : MLPNTGKLAGCTVFITGASRGIGKAIALK
Probability of export to mitochondria: 0.9797

Fig.S2 - HSDL2 is a mitochondrial protein. (A) Schematic presentation of HSDL2 conservation between species. This figure was generated using the Constraint-based Multiple Alignment Tool (COBALT), a multiple sequence alignment tool that finds a collection of pairwise constraints derived from conserved domain database, protein motif database, and sequence similarity, using RPS-BLAST, BLASTP, and PHI-BLAST. **(B-C)** Analysis of the N-terminal region of **(B)** human HSDL2 and **(C)** mouse HSDL2 for the identification of mitochondrial export signal using a method described by Claros *et al (36)*.

Figure S3 - The expression of *Hsdl2* is induced by PGC1α-PPARα.

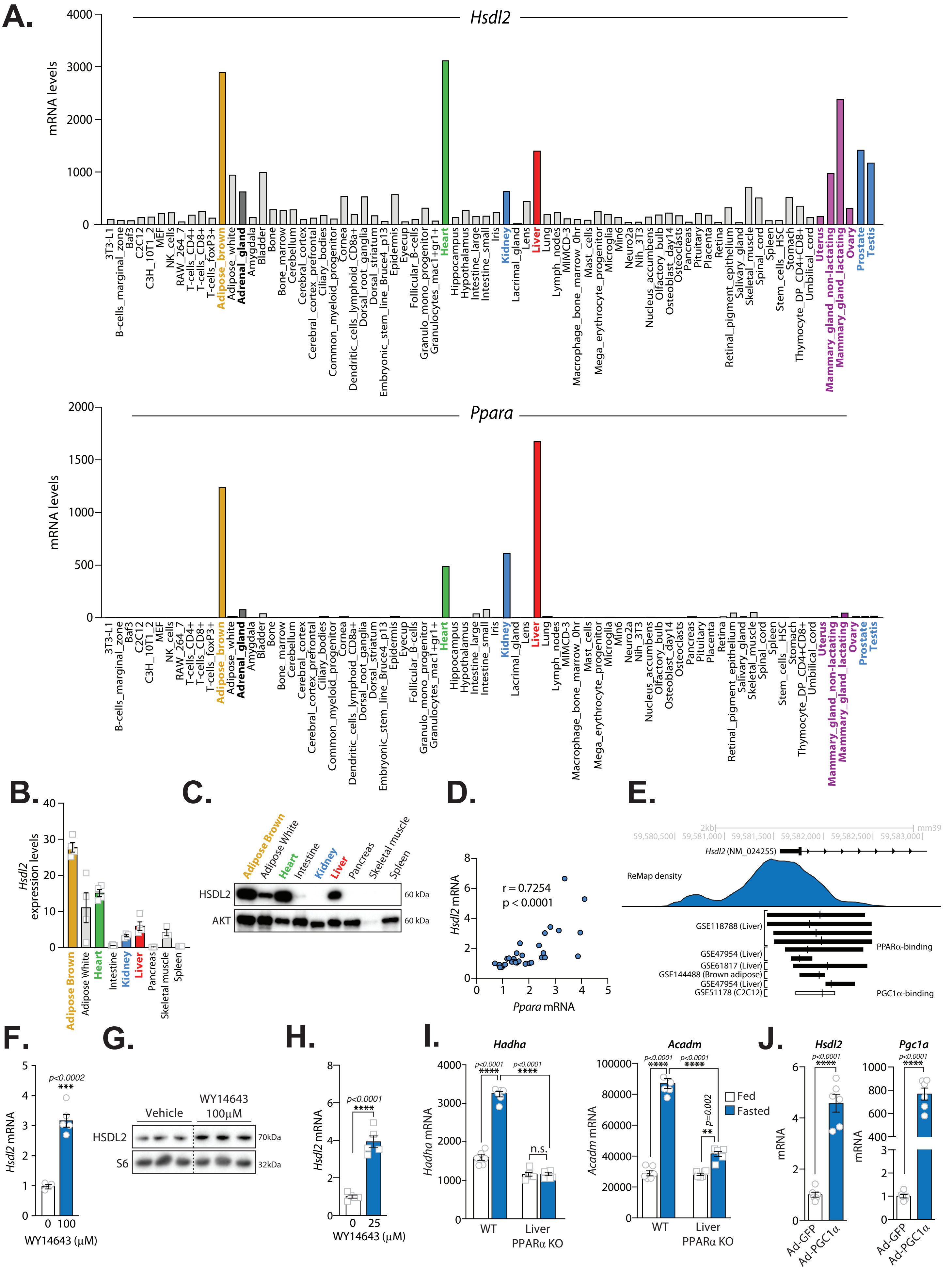

Fig.S3 - The expression of *Hsdl2* is induced by PPARα. (A) *Hsdl2* and *Ppara* expression profile in mouse tissues generated with BioGPS. **(B)** RNA was extracted from various mouse tissues and *Hsdl2* expression was quantified by RT-qPCR (n=4/tissue). Data are presented as mean ± SEM. **(C)** Proteins were extracted from various mouse tissues and HSDL2 protein expression was measured by western blot. Representative samples are shown. **(D)** Male mice were sacrificed after either 0, 6, 12 or 24 hours (n=8/group) of fasting and liver samples were collected. RNA was extracted and *Hsdl2* and *Ppara* expression was measured by RT-qPCR. The correlation between *Hsdl2* and *Ppara* mRNA is presented. **(E)** Analyses of quality-controlled mouse ChIP-Seq experiments showing the binding of PPARα and PGC1α to the proximal promoter of mouse *Hsdl2*. This analysis was generated using the ReMap resource described by Hammal *et al*. *(40)* **(F)** FAO cells were treated for 6 hours with vehicle (DMSO) or WY14643 at the indicated dose. RNA was extracted and *Hsdl2* expression was quantified by RT-qPCR (n=4). **(G)** Protein lysates were prepared from the experiment described in F and western blots were performed for the indicated proteins (n=3/group). **(F)** Primary hepatocytes were treated for 6 hours with vehicle (DMSO) or WY14643 at the indicated dose. RNA was extracted and *Hsdl2* expression was quantified by RT-qPCR (n=5). **(I)** The publicly available NCBI's Gene Expression Omnibus (GEO) dataset GSE96559 was used for this analysis *(41)*. Results obtained for *Hadha* and *Acadm* with this microarray analysis are presented. **(J)** Adenoviruses driving overexpressing of PGC1α1 or a control (Vector) were tail-vein injected in C57BL6J mice and the liver was collected 7 days post-injection. *Hsdl2* and *Pgc1a* mRNA expression was measured by RT-qPCR in these samples (n=6 per group). Except for panel A, all data are presented as mean ± SEM. In panel F, H and J, significance was determined by two tailed, unpaired *t* test. In panel I, significance was determined by two-way ANOVA with Tukey's multiple-comparisons test.

Figure S4 - The expression of *Hsdl2* is repressed by insulin downstream of mTORC1.

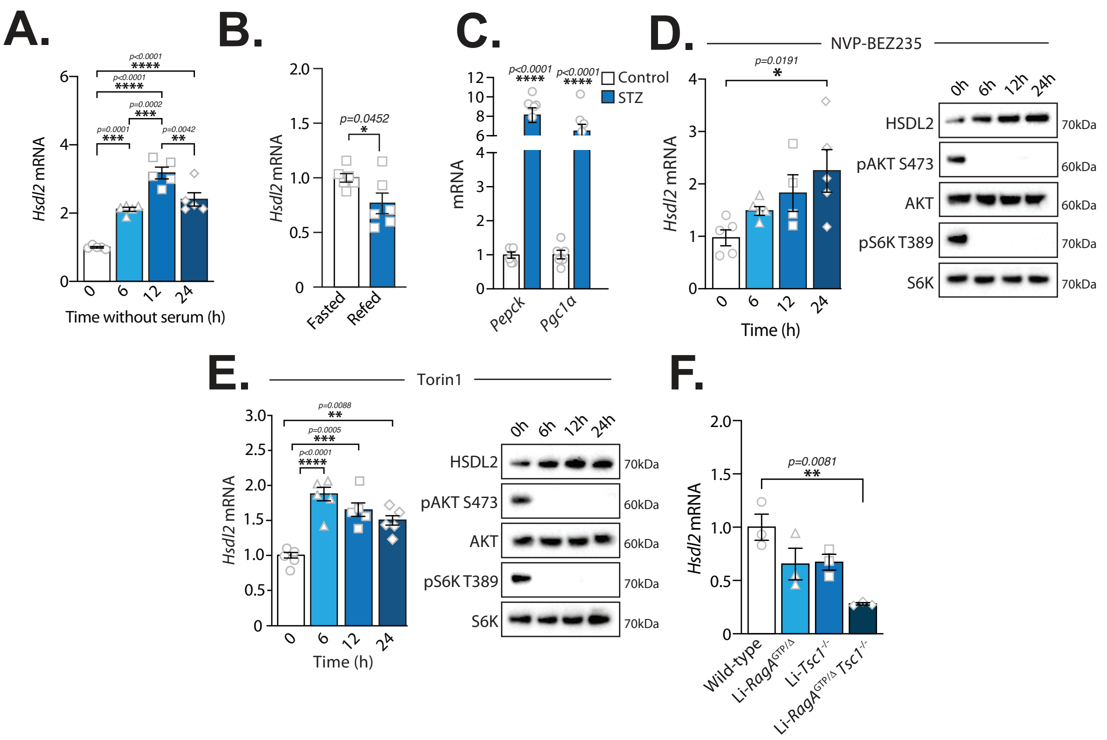

Fig.S4 - The expression of *Hsdl2* is repressed by insulin downstream of mTORC1. (A) FAO cells were serum-deprived for 6, 12, or 24 hours. RNA was extracted and *Hsdl2* expression was measured by RT-qPCR (n=5/group). **(B)** Mice were fasted for 24 hours and then refed normal chow for 2 hours. RNA was extracted from liver samples and *Hsdl2* expression was measured by RT-qPCR (n=6/group). **(C)** The mice used in the experiment described in Figure 4D were sacrificed 7 days post-injection and liver samples were collected. RNA was extracted and gene expression was measured by RT-qPCR (n=6-8/group). **(D)** FAO cells were treated with NVP-BEZ235 (250nM) for 6, 12, or 24 hours. Left panel: RNA was extracted and *Hsdl2* expression was measured by RT-qPCR (n=4-5/condition). Right panel: protein lysates were extracted, and western blots were performed for the indicated proteins. **(E)** FAO cells were treated with Torin1 (250nM) for 6, 12, or 24 hours. Left panel: RNA was extracted and *Hsdl2* expression was measured by RT-qPCR (n=5/condition). Right panel: protein lysates were extracted, and western blots were performed for the indicated proteins. **(F)** Mice of the indicated genotypes were fasted for 24 hours and sacrificed. RNA was extracted from liver samples and *Hsdl2* expression was measured by RT-qPCR (n=3/group). In all panel, data are presented as mean ± SEM. In panel A, D, E, and F, significance was determined by one-way ANOVA with Tukey's multiple-comparisons test. In panel B and C, significance was determined by two tailed, unpaired *t* test.

Figure S5 - HSDL2 depletion impairs mitochondrial respiration and metabolism.

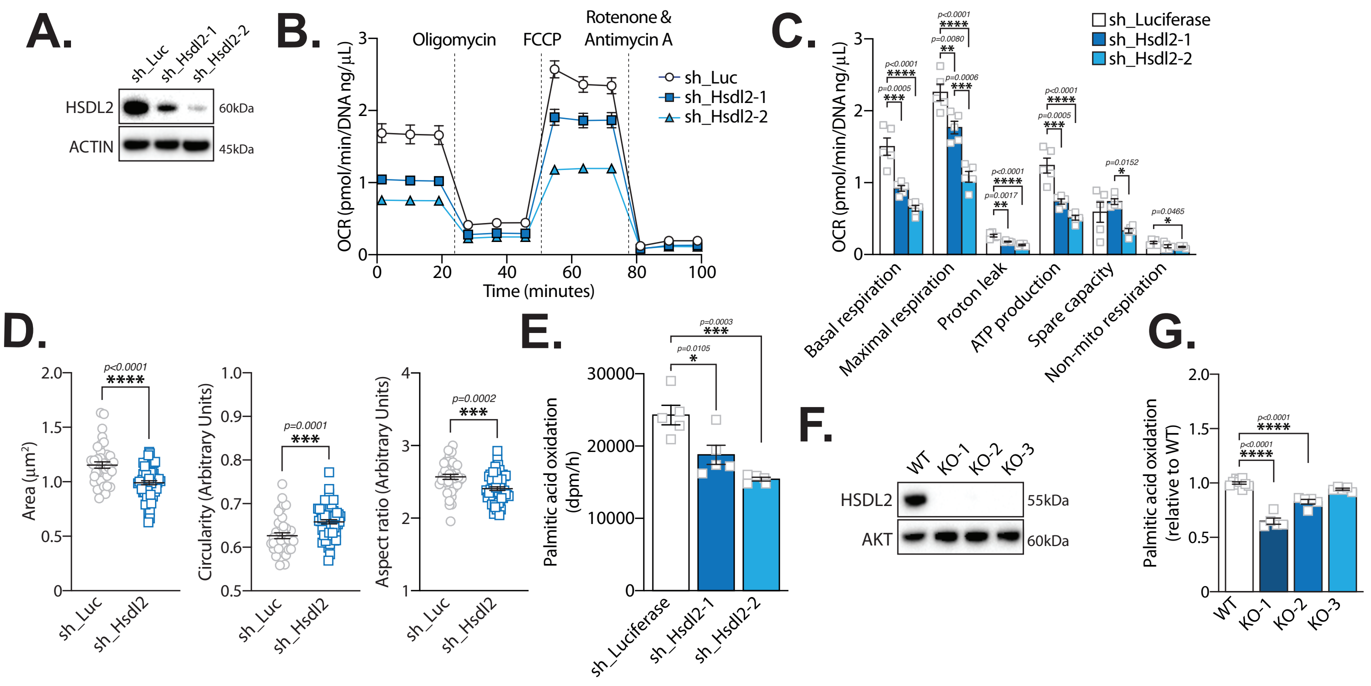

Fig.S5 - HSDL2 depletion impairs mitochondrial respiration and metabolism. **(A)** Hepa 1-6 cells were transduced with lentivirus expressing either a control shRNA (sh_Luciferase) or shRNA targeting *Hsdl2* (sh_Hsdl2-1 or sh_Hsdl2-2) and cells were selected with puromycin. Proteins were extracted seven days post-selection and western blot was performed. **(B)** Oxygen consumption rate measurement in Hepa 1-6 cells during the Seahorse Mitostress protocol (n=5 per condition). **(C)** Respiration parameters calculated from the graph presented in B. **(D)** Mitochondrial morphology parameters (area, circularity, and aspect ratio) were calculated in HepG2 cells expressing either sh_Luc or sh_Hsdl2. **(E)** Measurement of palmitic acid oxidation in Hepa 1-6 cells with or without HSDL2 knockdown. **(F)** HEK293 cells were transfected with one sgRNA targeting HSDL2 and were selected with puromycin. A clonal selection was performed and HSDL2 loss was validated by western blot. **(G)** Measurement of palmitic acid oxidation in the HSDL2 KO clones prepared as described in F. In all panels, data are presented as mean ± SEM. In panel C, E, and G, significance was determined by one-way ANOVA with Tukey's multiple-comparisons test. In panel D, significance was determined by two tailed, unpaired *t* test.

Figure S6 - HSDL2 controls cholesterol conversion to bile acids and FXR activation.

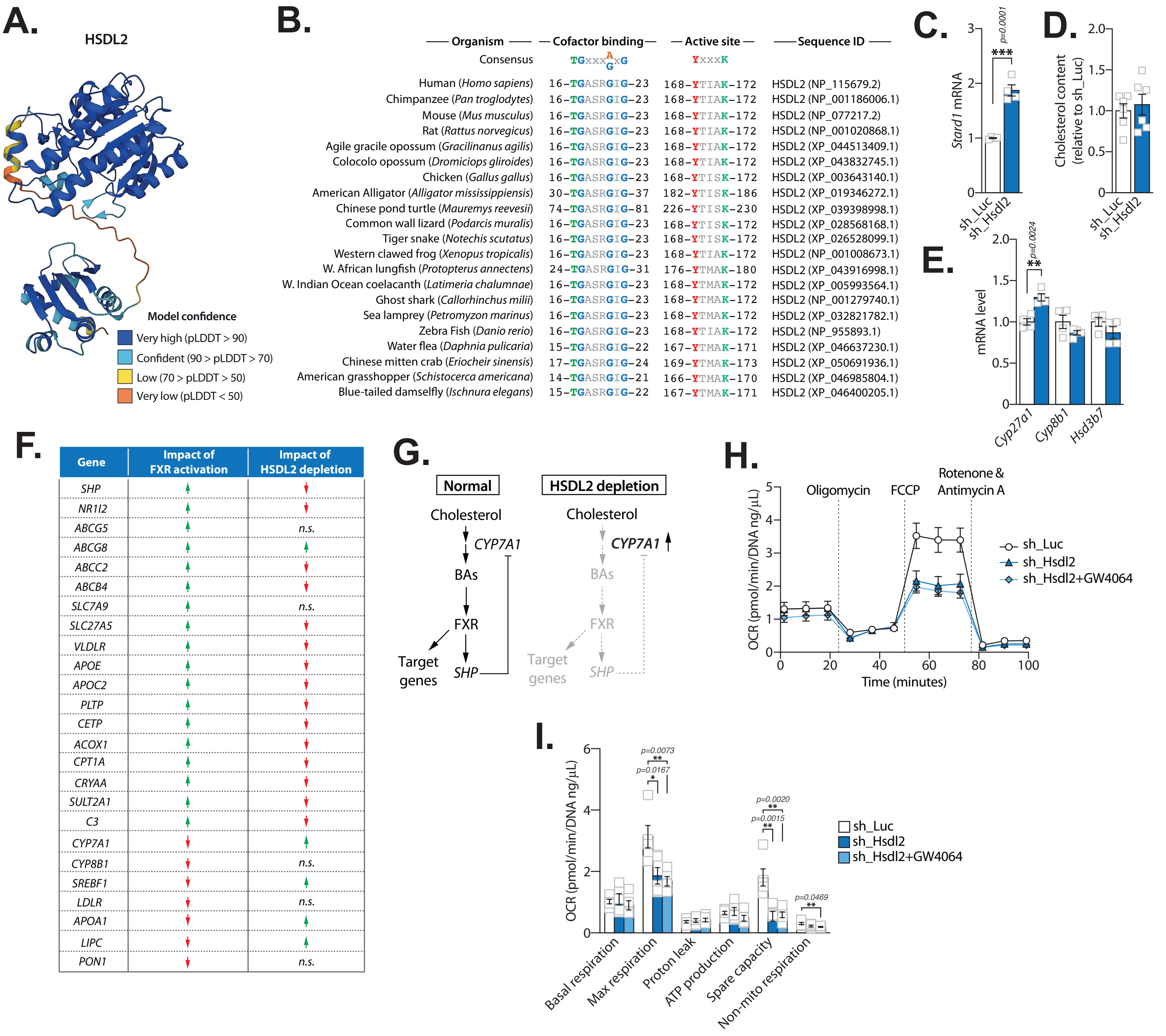

Fig.S6 - HSDL2 controls cholesterol conversion to bile acids and FXR activation. (A) HSDL2 structure modelized with AlphaFold. **(B)** Conservation of the cofactor binding sequence and the active catalytic site in HSDL2 between species. **(C)** HepG2 cells were infected, selected, and amplified. RNA was extracted and the expression of *Stard1* was measured by RT-qPCR (n=4/condition). **(D)** Mitochondrial cholesterol content measured after 24 hours of incubation with radiolabeled [^{3}H]-Cholesterol (n=6/group). **(E)** mRNA expression of *Cyp27a1*, *Cyp8b1*, and *Hsd3b7* in HepG2 following *Hsdl2* knockdown (n=4/condition). **(F)** Table presenting the classical FXR target genes in human cells. On the left side of the panel, the directionality of the changes associated with FXR activation is presented. On the right side, the impact of HSDL2 knockdown on the expression of these genes is presented. These results are related to Figure 6D. **(G)** Schematic description of *CYP7A1* regulation in normal conditions and in response to HSDL2 depletion. **(H)** HepG2 cells were exposed to a vehicle (DMSO) or GW4064 for 24 hours. Oxygen consumption rate was then measured with the Seahorse Mitostress protocol (n=5/condition). **(I)** Respiration parameters calculated from the graph presented in F. In all panels, data are presented as mean ± SEM. In panel C, D, and E, significance was determined by two tailed, unpaired *t* test. In panel I, significance was determined by one-way ANOVA with Tukey's multiple-comparisons test.

Figure S7 - Liver-specific HSDL2 depletion impacts hepatic BA metabolism and circulating cholesterol in mice.

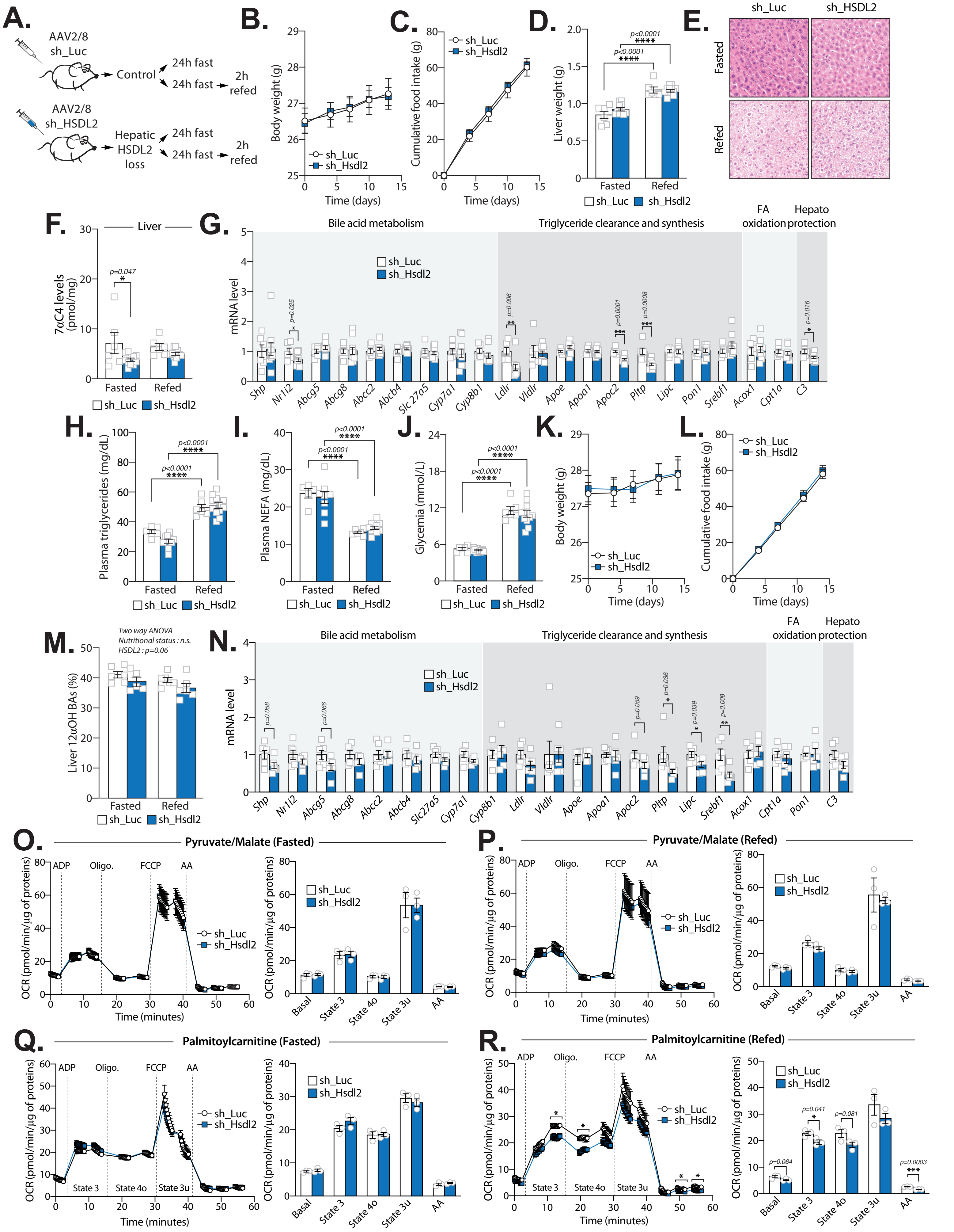

Fig.S7 - Liver-specific HSDL2 depletion impacts hepatic BA metabolism and circulating cholesterol in mice. (A) Representation of the experimental scheme performed in mice to study the impact of *Hsdl2* knockdown in the liver. Male mice were used in all the studies. **(B)** Body weight **(C)** and cumulative food intake of control and liver-specific HSDL2 knockdown mice two weeks following the AAV2/8 injection (n=12 for AAV2/8-sh_Luc, n=22 for AAV2/8-sh_Hsdl2). These mice were fed a chow diet. **(D)** Liver weight of control and liver-specific HSDL2 knockdown mice fed chow at sacrifice, 2 weeks following the AAV2/8 injection (Fasted: n=6 for AAV2/8-sh_Luc, n=10 for AAV2/8-sh_Hsdl2; Refed: n=6 for AAV2/8-sh_Luc, n=12 for AAV2/8-sh_Hsdl2). **(E)** Hematoxylin and eosin-stained sections of liver samples collected from control and liver-specific HSDL2 knockdown mice fed chow diet. Representative pictures are shown. **(F)** AAV2/8-sh_Luc and AAV2/8-sh_Hsdl2 mice were sacrificed following either a fasting or a refeeding period (Fasted: n=6 for AAV2/8-sh_Luc, n=10 for AAV2/8-sh_Hsdl2; Refed: n=6 for AAV2/8-sh_Luc, n=12 for AAV2/8-sh_Hsdl2). Levels of 7αC4 were measured in the liver. **(G)** AAV2/8-sh_Luc and AAV2/8-sh_Hsdl2 mice were sacrificed after 24 hours of fasting and liver samples were collected (n=6 for AAV2/8-sh_Luc, n=10 for AAV2/8-sh_Hsdl2). RNA was extracted and gene expression was measured by RT-qPCR. **(H)** Plasma triglycerides, **(I)** NEFA, and **(J)** insulin levels were measured in control and liver-specific HSDL2 knockdown mice after a fasting or refeeding period (Fasted: n=6 for AAV2/8-sh_Luc, n=10 for AAV2/8-sh_Hsdl2; Refed: n=6 for AAV2/8-sh_Luc, n=12 for AAV2/8-sh_Hsdl2). **(K)** Body weight and **(L)** cumulative food intake of control and liver-specific HSDL2 knockdown mice two weeks following the AAV2/8 injection (n=12 for AAV2/8-sh_Luc, n=11 for AAV2/8-sh_Hsdl2). These mice were fed high cholesterol diet. **(M)** Percentage of 12α hydroxylated BAs over total BAs in the liver of AAV2/8-sh_Luc and AAV2/8-sh_Hsdl2 mice after a fasting or refeeding period. These mice were fed a high cholesterol diet. (Fasted: n=6 for AAV2/8-sh_Luc, n=5 for AAV2/8-sh_Hsdl2; Refed: n=6 for AAV2/8-sh_Luc, n=6 for AAV2/8-sh_Hsdl2). **(N)** AAV2/8-sh_Luc and AAV2/8-sh_Hsdl2 mice were fasted and refed a high cholesterol diet for 2 hours. RNA was extracted and gene expression measured by RT-qPCR (n=6 for AAV2/8-sh_Luc, n=6 for AAV2/8-sh_Hsdl2). RNA was extracted and gene expression was measured by RT-qPCR. **(O)** Oxygen consumption rate in mitochondria extracted from the liver of 24h fasted AAV2/8-sh_Luc and AAV-sh_Hsdl2 mice fed a high cholesterol diet (n=3 for AAV2/8-sh_Luc, n=3 for AAV2/8-sh_Hsdl2). Pyruvate and malate were added at the beginning of the assay to provide substrates to the complex I of the electron transport chain. (**P)** Oxygen consumption measurement in mitochondria extracted from the liver of 2h refed AAV2/8-sh_Luc and AAV2/8-sh_Hsdl2 mice fed a high cholesterol diet (n=3 for AAV2/8-sh_Luc, n=3 for AAV2/8-sh_Hsdl2). Pyruvate and malate were added at the beginning of the assay to provide substrates to the complex I of the electron transport chain. **(Q)** Oxygen consumption measurement in mitochondria extracted from the liver of 24h fasted AAV2/8-sh_Luc and AAV2/8-sh_Hsdl2 mice fed a high cholesterol diet (n=3 for AAV2/8-sh_Luc, n=3 for AAV2/8-sh_Hsdl2). Palmitoylcarnitine was injected with ADP to assess fatty acid oxidation. **(R)** Oxygen consumption measurement in mitochondria extracted from the liver of 2h refed AAV2/8-sh_Luc and AAV2/8-sh_Hsdl2 mice fed a high cholesterol diet (n=3 for AAV2/8-sh_Luc, n=3 for AAV2/8-sh_Hsdl2). Palmitoylcarnitine was injected with ADP to assess fatty acid oxidation. In all panels, data are presented as mean ± SEM. In panel D, F, H, I, J and M, significance was determined by two-way ANOVA with Tukey's multiple-comparisons test. In panel G, N, O, P, Q and R significance was determined by two tailed, unpaired *t* test.

Table S1. Gene expression profiling of Low and High glucose-producing lines. Microarray analyses were performed in Low (n=6) and High (n=6) catabolic cell lines. Results are presented as fold change (High vs Low). This table is provided as an Excel file.

Table S2. Metascape analysis presenting the ontology parent pathways differing between Low and High lines. The genes differentially expressed between Low and High lines listed in Table S1 were analyzed using Metascape to identify the parent pathways enriched. The results of this analysis are presented in Fig.S1C. This table is provided as an Excel file.

Table S3. Metascape analysis presenting the cellular processes differing between Low and High lines. The genes differentially expressed between Low and High lines listed in Table S1 were analyzed using Metascape to identify the cellular processes enriched. The results of this analysis are presented in Fig.S1D. This table is provided as an Excel file.

Table S4. Scoring matrix used to prioritize candidate genes linked to catabolism in hepatocytes. The list of 608 genes differentially expressed between the Low and High lines was extracted and filtered to isolate the most differentially expressed genes (fold change below 0.7 or above 1.3). This analysis reduced the list of candidates to 106 genes. These genes were next scored for i) the pattern of expression during fasting, ii) the intensity of gene expression change during fasting, iii) the consistence of gene regulation between *in vivo* and *in vitro* data, and iv) the tissue distribution in mice. Here comes a description of each criterion. *Pattern of expression in the liver upon fasting:* Each gene was scored for its pattern of expression in liver upon fasting based on data published by Hakvoort et al *(7)*. A score of 1 was given to the genes showing change upon fasting (increase, decrease, or biphasic modulation). A score of 0.5 was given to the genes for which no data was available. A score of 0 was given to genes that showed no regulation upon fasting. This analysis was key to prioritize genes regulated by fasting. *Intensity of gene expression change upon fasting:* Each gene was scored for the magnitude of its regulation upon fasting based on data published by Hakvoort et al *(7)*. A scale of 0 to 1 was used. This analysis was key to prioritize genes whose expression was the most affected by fasting. *Consistence of gene regulation between in vivo and in vitro data:* We next scored the consistency of gene regulation *in vivo* and *in vitro* by comparing the directionality of expression of each candidate in mouse liver upon fasting to the expression pattern in the High lines. Genes increased upon fasting and enriched in High catabolic lines were given a score of 1. Genes decreased upon fasting and reduced in High catabolic lines were also given a score of 1. Genes that did not follow these patterns were given a score of 0. This analysis was key to prioritize genes whose regulation was consistent between different catabolic contexts. *Tissue distribution in mice:* We next scored candidate gene based on their expression levels in mouse tissues/cell lines using BioGPS *(37, 38)*. Genes showing an expression level in the liver that was 3 times above the median expression of other tissues/cell types were given a score of 1. This analysis was key to prioritize genes whose expression was enriched in the liver. This table is provided as an Excel file.

Table S5. List of genes correlating with *Hsdl2* in mouse tissues/cell types. Genes correlating with *Hsdl2* in mouse tissues/cell types were identified using BioGPS *(37, 38)*. The correlation cutoff was arbitrary fixed at 0.7. Correlation coefficients are presented for various probes

(reporters). The unified list of genes correlating with *Hsdl2* is presented on the right side of Table S5. This table is provided as an Excel file.

Table S6. Metascape analysis presenting the cellular processes enriched from the genes correlating with *Hsdl2* in mouse tissues/cell types. The genes correlating with *Hsdl2* (Table S5) were analyzed using Metascape to identify the cellular processes enriched in this data set. The results of this analysis are presented in Fig.3A. This table is provided as an Excel file.

Table S7. Bile acid profiling in the liver of control and liver-specific HSDL2 knockdown mice. Bile acid levels in the liver of control and liver-specific HSDL2 knockdown mice at sacrifice, 2 weeks following the AAV2/8 injection (Fasted: n=6 for AAV2/8-sh_Luc, n=10 for AAV2/8-sh_Hsdl2; Refed: n=6 for AAV2/8-sh_Luc, n=12 for AAV2/8-sh_Hsdl2). Results are presented as pmol/mg of tissue. Data are presented as mean ± SEM. For all BA species, significance was determined by two tailed, unpaired *t* test on log transformed data. This table is provided as an Excel file.

Table S8. Bile acid profiling in the plasma of control and liver-specific HSDL2 knockdown mice. Bile acid levels in the plasma of control and liver-specific HSDL2 knockdown mice at sacrifice, 2 weeks following the AAV2/8 injection (Fasted: n=6 for AAV2/8-sh_Luc, n=10 for AAV2/8-sh_Hsdl2; Refed: n=6 for AAV2/8-sh_Luc, n=12 for AAV2/8-sh_Hsdl2). Data are presented as mean ± SEM. Results are presented as nmol/L. For all BA species, significance was determined by two tailed, unpaired *t* test on log transformed data. This table is provided as an Excel file.

Table S9. Bile acid profiling in feces of control and liver-specific HSDL2 knockdown mice. Bile acid levels in feces of control and liver-specific HSDL2 knockdown mice at sacrifice, 2 weeks following the AAV2/8 injection (Fasted: n=6 for AAV2/8-sh_Luc, n=10 for AAV2/8-sh_Hsdl2; Refed: n=6 for AAV2/8-sh_Luc, n=12 for AAV2/8-sh_Hsdl2). Data are presented as mean ± SEM. Results are presented as pmol/mg. For all BA species, significance was determined by two tailed, unpaired *t* test on log transformed data. This table is provided as an Excel file.

Table S10. Bile acid profiling in the liver of control and liver-specific HSDL2 knockdown mice fed a high cholesterol diet. Bile acid levels in the liver of control and liver-specific HSDL2 knockdown mice at sacrifice, 2 weeks following the AAV2/8 injection (Fasted: n=6 for AAV2/8-sh_Luc, n=5 for AAV2/8-sh_Hsdl2; Refed: n=6 for AAV2/8-sh_Luc, n=6 for AAV2/8-sh_Hsdl2). Results are presented as pmol/mg of tissue. Data are presented as mean ± SEM. For all BA species, significance was determined by two tailed, unpaired *t* test on log transformed data. This table is provided as an Excel file.

Table S11 Bile acid profiling in the plasma of control and liver-specific HSDL2 knockdown mice fed a high cholesterol diet. Bile acid levels in the plasma of control and liver-specific HSDL2 knockdown mice at sacrifice, 2 weeks following the AAV2/8 injection (Fasted: n=6 for AAV2/8-sh_Luc, n=5 for AAV2/8-sh_Hsdl2; Refed: n=6 for AAV2/8-sh_Luc, n=6 for AAV2/8-sh_Hsdl2). Data are presented as mean ± SEM. Results are presented as nmol/L. For all BA

species, significance was determined by two tailed, unpaired *t* test on log transformed data. This table is provided as an Excel file.

Table S12. Bile acid profiling in feces of control and liver-specific HSDL2 knockdown mice fed a high cholesterol diet. Bile acid levels in feces of control and liver-specific HSDL2 knockdown mice at sacrifice, 2 weeks following the AAV2/8 injection (Fasted: n=6 for AAV2/8-sh_Luc, n=5 for AAV2/8-sh_Hsdl2; Refed: n=6 for AAV2/8-sh_Luc, n=6 for AAV2/8-sh_Hsdl2). Data are presented as mean ± SEM. Results are presented as pmol/mg. For all BA species, significance was determined by two tailed, unpaired *t* test on log transformed data. This table is provided as an Excel file.

Table S13. List of primers used in this study. This table is provided as an Excel file.
